# Virus-derived small RNAs in the penaeid shrimp *Fenneropenaeus chinensis* during acute infection of the DNA virus WSSV

**DOI:** 10.1038/srep28678

**Published:** 2016-06-28

**Authors:** Chengzhang Liu, Fuhua Li, Yumiao Sun, Xiaojun Zhang, Jianbo Yuan, Hui Yang, Jianhai Xiang

**Affiliations:** 1 Key Laboratory of Experimental Marine Biology, Institute of Oceanology, Chinese Academy of Sciences, Qingdao 266071, China; 2 Laboratory for Marine Biology and Biotechnology, Qingdao National Laboratory for Marine Science and Technology, Qingdao 266071, China

## Abstract

Small interfering RNAs (siRNAs) and microRNAs (miRNAs) are two classes of small RNAs (sRNAs) that are critical for virus-host interplay via the RNA interference (RNAi) pathway. One virus-derived siRNA and numerous miRNAs has been reported for the double-stranded DNA virus *white spot syndrome virus* (WSSV), however, the expression profiles of these different types of sRNAs have not been assessed. Here, by sequencing the sRNAs and mRNAs of WSSV-infected Chinese shrimp (*Fenneropenaeus chinensis*), we found that the viral transcripts were universally targeted by WSSV-derived siRNAs, supporting a pivotal role for RNAi in the anti-viral immunity of shrimp. The genesis of WSSV-derived siRNAs was associated with long RNA structures. Moreover, by separating miRNAs from siRNAs, 12 WSSV miRNAs were identified. Investigation of conserved viral miRNA targets in different host species indicated the involvement of viral miRNAs in host immune responses. Collectively, our data provide new insights into the role of the RNAi pathway in the interplay between DNA viruses and crustaceans.

The RNA interference (RNAi) pathway degrades mRNAs or inhibits their translation via gene silencing. Silencing occurs through RNA-induced silencing complexes (RISCs) consisting of an Argonaute protein (AGO) and a guiding small RNA (sRNA) that is antisense to the mRNA target^1^. In eukaryotes ranging from yeasts to humans, RNAi is an evolutionarily conserved mechanism that regulates gene expression and protects against viruses and transposons. Three classes of sRNAs are involved in RNAi: microRNAs (miRNAs) with a length of 18–24 nt, small interfering RNAs (siRNAs) of 21–23 nt, and PIWI-interacting RNAs (piRNAs) of 24–31 nt. These types of sRNAs guide the RNAi process in similar ways but employ different AGO proteins^2^.

The origins of siRNA and miRNA differ. miRNA biogenesis starts in the nucleus, wherein a primary miRNA (pri-miRNA) is transcribed and processed into shorter hairpin RNAs (precursor miRNAs or pre-miRNAs) by Drosha. A pre-miRNA is then exported to the cytoplasm and processed by Dicer to produce miRNA duplexes^3^. However, in most animal groups, siRNAs originate in the cytoplasm, wherein endogenous or exogenous dsRNAs are cleaved by Dicer^2^. In *Drosophila*, subsequent to the activity of Dicer, the RNAi pathways of sRNA duplexes from two different origins intersect: duplexes with central mismatches are sorted for AGO1, whereas highly paired duplexes are loaded into AGO2, thus forming different RISC complexes^4^,^5^.

Previous studies conducted in shrimp have identified components of the RNAi pathway, such as Drosha, Pasha, TRBP, Dicer1, Dicer2, AGO1, AGO2, and AGO3, revealing an RNAi system similar to that of insects^6^,^7^,^8^,^9^. Concomitantly, many shrimp miRNAs have been studied^10^, with an emphasis on those involved in innate immunity against viruses, such as miR-100^11^,^12^,^13^. To date, no reports have described shrimp piRNAs, perhaps due to the lack of available shrimp genome data.

Virus infection may induce the production of virus-derived small RNAs (vsRNAs) including siRNAs, miRNAs and/or piRNAs in diverse animals^14^. Various viruses express miRNAs to manipulate their own gene expression or that of the host to facilitate their life cycles. On the other hand, RNAi provides vital anti-viral defense for the host^14^. In this defense, viral dsRNA is recognized by Dicer2 and cleaved into vsiRNAs (virus-derived small interfering RNAs)^15^. These vsiRNAs are then loaded into an RISC to guide slicing of the target viral RNAs by an AGO2 present in the complex. These vsiRNAs are characterized by continuous coverage of genomic regions that are distinct from the discrete distribution of miRNAs^16^,^17^,^18^. Anti-viral RNAi is particularly important for plants and animals that lack a protein-based adaptive immune system^19^. It has been suggested that due to evolutionary pressure from viruses, Dicer in insects has diversified into Dicer1 and Dicer2 to reduce competition for Dicer between endogenous pre-miRNAs and exogenous viral dsRNAs^20^. Moreover, dsRNA-specific Dicer2 and AGO2 have become the most rapidly evolving *Drosophila* genes during the evolutionary arms race between *Drosophila* and rapidly evolving viruses^21^. vsiRNA was originally thought to be confined to RNA viruses with dsRNA genomes or replication intermediates that are targeted by host Dicer. However, genome-wide siRNA production has recently been discovered in DNA viruses that infect plants and insects^17^,^18^,^22^,^23^,^24^,^25^. In shrimp, natural siRNAs have also been reported, but only for the vp28 gene of *white spot syndrome virus* (WSSV)^26^. To our knowledge, genome-wide viral siRNA production has not been documented in any crustacean to date.

WSSV is a distinct DNA virus that has both economic and academic significance. With the capability to infect a wide range of crustaceans, including shrimp, crab, and crayfish, WSSV is a major pathogen in shrimp aquaculture and has caused serious losses worldwide since the 1990s^27^. It differs profoundly from all presently known viruses and is the sole representative of the Nimaviridae family^28^,^29^. Its circular double-stranded DNA (dsDNA) genome is among the largest of all animal viruses (305 kb). One of the first animal DNA viruses shown to produce natural siRNAs^26^, it has also been reported to express numerous miRNAs^30^,^31^. However, for WSSV, natural siRNAs were only discovered for the vp28 gene^26^, and it has been suggested that small RNA deep sequencing may provide more detailed insights to this issue^32^. Moreover, although the deep-sequencing technique has the potential to discriminate between miRNAs and siRNAs^33^, their differences have not been addressed in WSSV.

Thus, we aimed to determine whether WSSV genes other than vp28 are targeted by shrimp RNAi, and to clarify the proportions of miRNA and siRNA of WSSV. For these purposes, we conducted an RNA-seq study of sRNAs and mRNAs from the Chinese Shrimp (*Fenneropenaeus chinensis*) during latent infection (LI) and acute infection (AI) with WSSV. To identify the origin of viral sRNAs, the sRNAs were compared to the genome and mRNAs of WSSV. Bioinformatic analyses were then carried out to classify the viral sRNAs (Fig. 1).

## Results

### Validation of LI and AI in the shrimp

In pleopod DNA from cephalothorax samples, the WSSV copy number was 119 times higher in AI shrimp than in LI shrimp ((2.93 ± 1.37) × 10^5^/ng and (2.46 ± 0.23) × 10^3^/ng, respectively, n = 10 each). Consistent with the DNA data, the mRNA diversity and expression also increased dramatically in WSSV, with 111 transcripts detected during the AI stage and none detected during the LI stage^34^.

### Genome-wide viral sRNA production during acute infection

After removing sRNAs that aligned with host mRNAs, the LI cephalothorax sample provided 1,664 unique sRNAs that aligned with the WSSV genome. During the AI stage, the expression of WSSV-derived sRNA increased abruptly by 188-fold (Table 1).

There were 545 unique WSSV-derived sRNAs found exclusively in LI cephalothoraxes, while 130,518 were found only in the AI stage. Additionally, 1119 viral sRNAs were present in both stages, accounting for 67% of the viral sRNAs in the LI stage but only 0.85% of those in the AI stage. The accumulated expression of these overlapping sRNAs was up-regulated 61-fold during the AI stage. None of the viral sRNAs detected in the LI stage were significantly down-regulated during the AI stage.

Viral sRNAs of 18–35 nt in length were found to be ubiquitously distributed along the WSSV genome (Fig. 2C). In the figures, we refer to the viral strand that is transcribed from left to right as the plus strand and to its complement as the minus strand. A distinct sRNA hotspot was identified at 151 kb–157.5 kb on the minus strand. The strand-specific density of unique sRNAs across the genome provided an average value of 0.22 (Table 1). Thus, on average, an unique sRNA was found on either strand at intervals no larger than 5 nt. The majority (82%) of the 305-kb viral genome was encompassed by sRNAs on either the plus (55%) or minus (58%) strand, and the sequencing depth per nucleotide ranged from 0–1,463 for the plus strand (average = 10) and from 0–35,587 (average = 31) for the minus strand.

### WSSV mRNAs were selectively processed to generate viral sRNAs

The ubiquitous production of viral sRNAs has raised the question of whether they are vsiRNAs or just normal mRNA degradation products. This was assessed from three perspectives.

First, the sRNA reads were classified to clarify their major components. The clean reads were annotated by comparison with the Rfam non-coding RNA database and the miRBase miRNA database. The majority of the sRNAs belonged to known sRNA categories (Supplementary Fig. S1). Considering that host piRNAs, endogenous siRNAs, and novel miRNAs cannot be identified based on sequence similarity, the proportion of genuine sRNAs should be even higher than the current findings. These results indicated that the majority of the total sRNAs were not degraded mRNAs.

Second, to evaluate whether the unidentified short RNAs were degradation products, they were mapped to protein coding sequences (CDSs) from the transcriptome data of the same samples (see Methods). If the mRNAs were degraded, both host and viral coding mRNAs should have produced abundant short RNA fragments. The sRNA distribution frequency in the WSSV CDSs (median = 141.9 reads per kilobase of transcript per million reads (RPKM)) was 53.2 times higher than that in the host CDSs (2.67 RPKM, *t*-test, P = 0; *F*-test, P = 0) (Fig. 3). This suggests that sRNAs were generated from viral mRNAs at productive ratios much higher than those from host mRNAs, which means viral sRNAs cannot be attributed to normal degradation of mRNAs. In other words, they originated from selective processing of viral transcripts.

Third, the distribution of sRNA lengths indicated that the WSSV-derived sRNAs were products of Dicer processing. The distinct enrichment of unique sRNAs with a length of 21–23 nt is a characteristic of Dicer processing (Fig. 4E,F), in stark contrast to the unique host sRNAs, which predominantly displayed lengths of 26–27 nt (Fig. 4A–C), a very small proportion of which were miRNAs (Supplementary Fig. S1).

In brief, the combined information for the length distribution and classification of sRNAs as well as the level of sRNA production from coding sequences suggested that the majority of WSSV-derived sRNAs were not degraded mRNAs but were, in fact, Dicer products.

### Relationship between the distribution of viral sRNAs and mRNAs

Because RNAi provides an anti-viral mechanism by selectively degrading viral mRNAs, which results in the production of viral siRNAs, a correlation between the genomic distribution of viral sRNAs and mRNAs is expected. In fact, regions that lacked mRNA expression usually also lacked sRNA production (Fig. 2B,C, Supplementary Fig. S2C,D), indicating that viral sRNAs were derived from mRNAs. A weak correlation was observed between the expression of viral sRNA and mRNA (Supplementary Fig. S3, R = 0.52), suggesting that factors other than the quantity of mRNA also affected the genesis of sRNA. The viral transcripts that matched the most sRNAs were located at ~155 kb in the virus genome, including wsv271 (VP136) and wsv277 (Fig. 5). A second sRNA hotspot residing at ~220 kb was represented by wsv360 (VP664). Hotspots for sRNAs of different lengths usually coincided in terms of the genomic location. However, at certain locations, their proportions were quite different, suggesting the presence of different classes of sRNAs (Fig. 2C, Supplementary Fig. S2 D).

### Genomic characteristics of WSSV-derived sRNAs

Whether WSSV-derived sRNAs were miRNAs, piRNAs or siRNAs was what we wanted to find out next.

We first evaluated the sRNAs for piRNA characteristics. piRNAs are generally longer than siRNAs or miRNAs^35^. Known characteristics of piRNAs include an overlap of 10 nt between the 5′ ends of the sense and antisense piRNAs, as well as a strong sequence bias for uridine at position 1 (1 U bias) in piRNAs bound by PIWI and a bias for adenosine at position 10 (10A bias) in piRNAs bound by AGO3^36^. However, the majority of WSSV-derived sRNAs did not display the above characteristics of piRNAs (Supplementary Figs S4 and S5). Moreover, the typical length of piRNAs is 24–31 nucleotides, but the WSSV-derived sRNAs were predominantly 21~23nt in length (Fig. 4E,F,K,L). This is a feature of Dicer products, indicating that the majority of sRNAs were either miRNAs or siRNAs.

The WSSV-derived sRNAs were then assessed for miRNA characteristics. Because a hairpin-like RNA structure is required for processing of pre-miRNAs by Dicer^1^,^2^, we scanned the non-coding region of the WSSV genome for pre-miRNAs and found 1,255 potential pre-miRNAs. Because miRNAs are generated from the stem regions of pre-miRNAs, the sRNA frequencies were compared between the potential pre-miRNA structures, the non-coding genomic regions, and the whole viral genome (Fig. 6). We found that sRNA frequency was significantly higher in the stems of hairpin structures than in other regions (Wilcoxon rank-sum test, W = 1.0 × 10^10^, P = 2.2 × 10^−16^), indicating the production of miRNAs. However, potential miRNAs (sRNAs generated from hairpin stems) made up only 3.6% of all the WSSV-derived sRNAs at the AI stage. Taken together, the above information suggested that viral miRNAs were produced during AI, although they only made up a minor portion of the WSSV-derived sRNAs.

Because miRNAs and piRNAs were deemed unlikely to comprise the major component of WSSV-derived sRNAs, the sRNAs was hypothesized to be primarily siRNAs. One distinction between siRNA and miRNA is that siRNAs can be generated from viral ORFs^23^, while viral miRNAs are usually generated from non-coding regions^37^,^38^. We aligned the viral sRNAs to the WSSV ORFs from the transcriptome data, and found that 78.0% of the total WSSV-derived sRNAs originated from protein-coding sequences. This suggested that most were not miRNAs but siRNAs. Our sequencing data is consistent with previously reported vp28 siRNAs^26^. For the AI stage, 1,817 sRNA reads mapped to the vp28 CDS region (Supplementary Fig. S6), and of these, 22 reads overlapped with the published vp28 probes (Supplementary Text S1). In addition, the WSSV-derived sRNAs showed much higher sequence diversity than the host sRNAs, which were mainly miRNAs. Although the WSSV-derived sRNAs made up only 1.4% of the total AI sRNAs, they accounted for 14% of the unique sRNAs. High sequence diversity is a characteristics of siRNAs, because unlike miRNAs, siRNA genesis is not confined to pre-miRNA structures.

Although DNA viruses lack a dsRNA intermediate that could serve as an siRNA precursor, there may be other sources of dsRNA. Genesis of virus-derived siRNA from overlapping transcripts has been proposed by Bronkhorst *et al*. in a DNA virus^23^. This explanation is consistent with the overlapping nature of the ORFs in both strands of the WSSV genome. However, unlike Bronkhorst *et al*., we did not identify a strong correlation for the siRNA density between the two strands of individual ORFs (Supplementary Fig. S7). This indicated that convergent transcripts were not the major source of WSSV–derived siRNAs. siRNAs can also be derived from hairpin structures via a pathway that comprises Dicer2, Argonaute 2 and Loquacious, yielding another potential source of viral siRNA^39^,^40^. Because the hairpin structures that generate siRNAs can be much longer than pre-miRNAs, we examined the association between the sRNA frequency and long RNA structures. The RNA structure of the 6.5-kb sRNA hotspot in the minus strand at 151 kb–157.5 kb in the WSSV genome was predicted using RNAfold (Fig. 7A). More than 60 percent (3,924) of the nucleotides were located in the double-stranded region. The WSSV-derived sRNAs from the sequencing data were mapped to the structures. The number of nucleotides in the predicted double strands was calculated for each sRNA, and their relationship to the sequencing counts for the same sRNA was plotted (Fig. 7B). sRNAs with more paired nucleotides tended to have higher copy numbers. Significant copy number differences were identified between sRNAs (average count = 12.0) with greater numbers (≥12) of paired nucleotides and those (average count = 7.5) with lower numbers (≤11) of paired nucleotides (t = 1.2 × 10^−9^, *t*-test with unequal sample sizes and unequal variances; W = 1.1 × 10^9^, P = 1.6 × 10^−5^, Wilcoxon rank-sum test). The same results were also found for sRNAs obtained from an independent shrimp intestine sample (Fig. 7C). Thus, the production of WSSV-derived sRNAs is related to long hairpin structures.

Briefly, the majority of WSSV-derived sRNAs were most likely siRNAs, but their genomic characteristics also suggested the presence of miRNAs as a minor component.

### Viral miRNA identification

Taking the existence of siRNA into consideration, we refined the procedure for miRNA identification to reduce false-positive results by excluding candidates that overlapped with adjacent sRNAs (see “classification of viral sRNAs” in the Methods). Fourteen WSSV miRNAs were predicted from the sequencing data. To confirm the expression of these viral miRNAs, RT-PCR assays were conducted using stem-loop RT primers specific for detection of mature miRNAs^41^ (a loop structure in the primer prevents its hybridization with miRNA precursors, Supplementary Dataset S1). The expression of 12 of the 14 candidates was confirmed (Fig. 8, Table 2). The hairpin structures of their pre-miRNAs were conserved in the 4 WSSV strains (Supplementary Fig. S8; Text S2). Ten of the miRNAs were found to be novel, whereas 2 overlapped with reported WSSV miRNAs: 11 nt of wssv-m5 overlapped with wssv-miR-N1^31^, and 16 nt of wssv-m4 overlapped with wssv-miR-211^30^.

Expression of the viral miRNA in shrimp stomach and lymphoid organ was examined by RT-PCR. The results showed that the expression of wssv-m1 and m12 were much higher than others (Fig. 8A). Expression of most of the viral miRNAs continued to increase up to 48 hours post injection (hpi) (Fig. 8B). The expression levels of wssv-m5 and m6 were significantly higher in the lymphoid organ than in the stomach at 24 h, while at 48 h, their tissue expression patterns were the opposite of those observed at 24 h. Clustering of the variations in miRNA expression suggested that wssv-m7, m10 and m11 had the most similar expression patterns.

### Viral miRNA target analysis

Twenty-two WSSV genes were found to be potentially regulated by viral miRNAs, among which 14 were targeted by multiple miRNAs, indicating potential co-regulation of these miRNAs (Supplementary Table S1). wssv-m1 had the most viral targets (11), followed by wssv-m6 (7 targets), indicating that these miRNAs might play pivotal roles in viral gene regulation. WSSV miRNAs and their predicted viral target genes were compared among the genomes of 4 different WSSV strains from the NCBI. All of the miRNAs and their target sites were conserved among the strains. A comparison of 2 samples (AI cephalothorax and AI intestine) from two independent WSSV outbreaks was also performed. The results showed that all of the miRNA sequences were conserved. Examination of the viral target sequences showed that some target sites were missing due to alternative splicing or lack of expression, but no nucleotide substitutions were found at the target sites.

For the host genes, preliminary prediction using miRanda^42^ recovered 2,656 mRNAs as potential viral miRNA targets, indicating a broad influence on host gene expression.

To screen out pivotal target genes in the virus-host interactions, evolutionary data were explored. Considering that WSSV can infect a wide range of hosts, the regulation of a pivotal target gene by WSSV miRNAs should be conserved in different host species. Therefore, we searched for shared target genes of WSSV miRNAs in three host species, including two Penaeidae shrimp (*F. chinensis* and *L. vannamei*) and a Palaemonidae shrimp (*Exopalaemon carincauda*). All three species are susceptible to WSSV infection. The results revealed that 66 target genes were shared by all three hosts (Supplementary Dataset S2; Fig. S9A), among which 22 had binding sites that were evolutionarily conserved (Supplementary Dataset S3; Fig. S9B).

Among the 22 host genes that contained conserved binding sites for WSSV miRNAs, 15 have been reported to be involved in innate immunity, the cell cycle, or other virus-related processes. The *PL10A/Belle/DDX3* dead-box helicase subfamily is associated with antiviral signaling and RNAi^43^, and it is targeted by diverse viruses for inhibition^44^. *p120*-catenin regulates a wide range of biological processes, including cell proliferation, inflammation and innate immunity^45^. The *14-3-3* gene participates in several signal transduction pathways, and it has been reported to be involved in anti-viral responses in shrimp^46^ and humans^47^. Eukaryotic translation initiation factor 5A (*eIF5A*) is implicated in cell cycle control and has been associated with WSSV infection^48^. The predicted regulation will be verified in future studies.

### Visualization of the viral miRNA regulation network

To gain a better understanding of the intricate relationships between viral miRNAs and their targets, the interactions were visualized with Cytoscape. Gene annotation and expression data were also visualized (Supplementary Fig. S9). As indicated by the symbol size, it is readily apparent that the most abundant WSSV miRNAs were wssv-m1 and m12, while the most abundant host target gene was MLR (myosin light chain 2). Host genes that were targeted by multiple miRNAs were placed in the middle of the network, which revealed that the most strongly down-regulated mRNAs (blue circles) were targeted by multiple viral miRNAs, indicating possible co-regulation. As indicated by the thick line, the strongest regulation of a host gene was predicted between wssv-m1 and CTND2 (Supplementary Fig. S9A). All of the WSSV genes were up-regulated during acute infection, as indicated in red (Supplementary Fig. S9C).

## Discussion

In recent years, virus-derived sRNAs (vsRNAs) have been detected in animals infected with various viruses^14^,^18^. Interestingly, the RNAi mechanisms associated with vsRNAs seems to be quite different between insect and mammalian hosts^49^. Supplementary Table S2 summarizes some recent studies of vsRNAs in animal viruses. In some cases, vsRNAs are ubiquitously and continuously produced, such that they can be assembled into long genomic contigs and even an entire viral genome^17^. Most of these discoveries were associated with RNA viruses. For DNA viruses, ubiquitous sRNA production has only been reported in insects^22^,^24^. The present study expands these findings to include a crustacean. In previous reports, the classification of vsRNAs was conducted according to their genomic characteristics and experimental evidence. In a few studies, piRNAs were identified in the presence of an abundance of 24–30-nt sRNAs displaying strong 5′ terminal uridine uridine bias and 10 nt adenine bias. In most of the other vsiRNA reports, viral sRNAs were classified as siRNAs because their continuous coverage of genomic regions was distinct from the discrete distribution of miRNAs^14^. In the cases of RVFV, VSV and WSSV, both virus-derived miRNAs and siRNAs were generated during the virus-host interaction. It is believed that viral miRNAs were encoded and expressed by the virus to regulate viral or host gene expression, while virus-derived siRNAs were generated via host RNAi response during which viral RNAs were detected and subjected for degradation^14^,^50^.

In the present study, adjacent siRNAs were considered during the identification of miRNAs, which has not yet been performed for WSSV. Following the miRbase protocol^51^, we successfully recovered known miRNAs (wssv-miR-211 and wssv-miR-N1) and siRNAs (for vp28). The biogenesis and functionality of these siRNAs and miRNAs have been experimentally validated previously^26^,^30^,^31^, supporting the reliability of our methods. Nevertheless, the sample types used in the sequencing studies here were limited. Additional sequencing studies should be carried out in the future using multiple tissue types at more infection stages, preferably from a different host species.

Consistent with most of the results of genome-wide viral sRNA analyses, the majority of WSSV-derived sRNAs were also suggested to be siRNAs. Canonical miRNAs also contributed to WSSV-derived sRNA production but only to a minor proportion of the sRNAs. Nonetheless, these viral miRNAs were dramatically up-regulated during the AI stage (Fig. 8), indicating their relevance to this stage. Although WSSV miRNAs were less abundant than WSSV-derived siRNAs, their amounts might still be present in sufficient quantities for target-regulation. Exploiting conserved miRNA targets from different host species enabled us to screen for important candidate targets of WSSV miRNAs, which indicated their roles in evading host innate immune responses such as antiviral signaling, inflammation and apoptosis. On the other hand, the autoregulatory WSSV miRNAs may contribute to the control of virus replication. These hypotheses should be verified in future studies.

One noteworthy characteristic of WSSV-derived sRNAs is their dramatic change in expression along the viral genome. The presence of sRNA hotspots has previously been reported for other viruses^23^,^24^,^52^. In this study, the presence of a distinct hotspots at 151.0~157.5 kb in the minus strand of the WSSV genome was confirmed in independent samples (Figs 2 and S2). Analysis showed that the production of sRNA from secondary structures of single-stranded RNAs (ssRNAs) might have produced the sRNA hotspot. This is consistent with previous studies which found that viral siRNAs can be derived from long hairpin structures via a pathway that comprises Dicer2, AGO2 and Loquacious^39^,^40^.

Our results expand the scope of WSSV–derived vsiRNA discovery from vp28 to the level of the whole genome. The findings of genome-wide siRNAs in a crustacean are consistent with the results obtained for insects. Taken together, these findings suggest that RNAi-mediated defense against DNA viruses may be common among arthropods. Studies of the functional role of siRNAs from individual WSSV genes should be carried out in the future, using mutant animals^23^ or by knocking down Dicer2 and/or AGO2^26^, as well as siRNA inhibitors^26^.

In contrast to viral infection of insect vectors, which show considerable resistance^53^, WSSV infection of the penaeid shrimp is virulent and usually lethal. This indicates that despite the degradation of viral mRNAs by host RNAi, which results in a large amount of viral siRNA production, WSSV can still overwhelm the host defenses, supporting the presence of viral countermeasures to the host RNAi system. The substantial co-expression of WSSV mRNAs with sRNAs during acute infection supports this speculation (Fig. 2). It has been proposed that a rapidly replicating virus can saturate the RNAi machinery^54^. However, if a virus can replicate so rapidly that viral RNA saturates the RNAi system, host RNAi should have already been futile against the virus prior to saturation. An alternative theory is that the virus uses junk RNAs as a decoy to divert the RNAi machinery so that the core part of the viral genome or transcripts achieve relative protection. This hypothesis is supported by the finding that hotspot vsiRNAs possess poor antiviral activity compared with coldspot vsiRNAs^52^. From a different perspective, Jayachandran *et al*. (2012) argued that the degradation of viral transcripts by host RNAi may be advantageous to the virus by preventing its over-replication, which may lead to the premature death of host cells^24^.

Previous studies have also identified various families of Viral Suppressors of RNA Silencing (VSR) proteins^19^. One VSR class, called siRNA sequesters binds to siRNAs and prevents their incorporation into RISC, which may protect viral mRNAs and promote the accumulation of viral siRNAs. This provides a potential explanation for the co-existence of WSSV mRNA and siRNA observed in the present study. We searched for homologs to siRNA sequesters in WSSV proteins, but we only identified sequences with low similarity. This was not unexpected because VSRs with similar functions can be strikingly diverse as a consequence of their independent evolution^19^. The discovery of genome-wide vsiRNAs in shrimp only raises questions regarding the complex mechanisms underlying their genesis and their roles in the context of RNAi against a DNA virus.

## Materials and Methods

**Figure 1:**
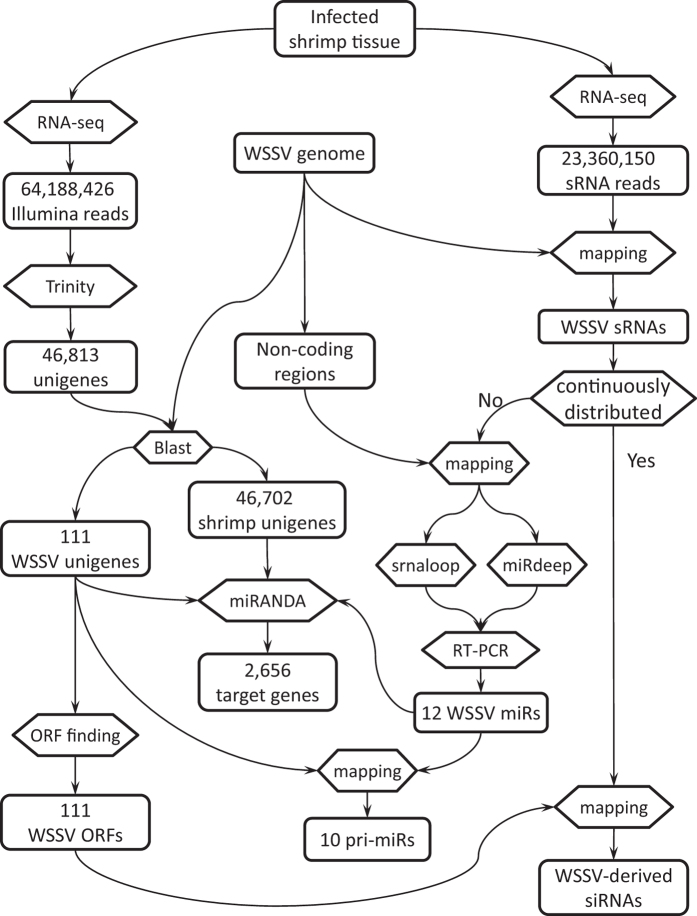
Roadmap of the bioinformatics analysis.

**Figure 2:**
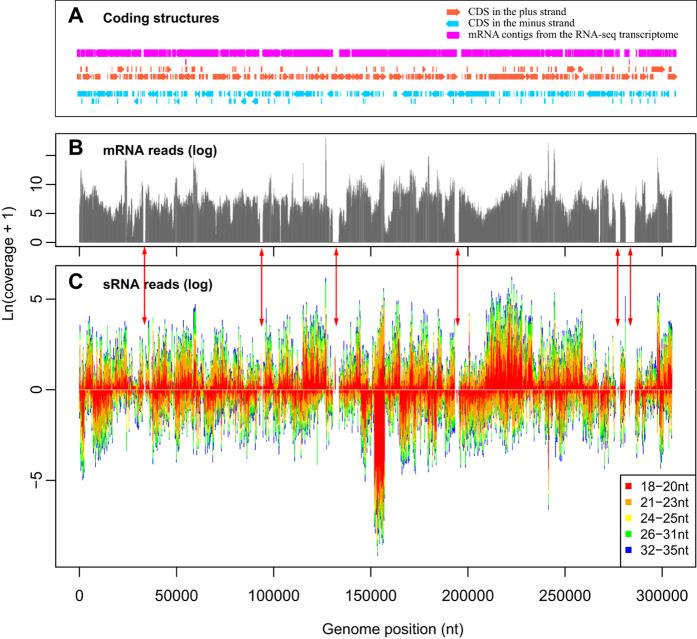
Distribution of sRNAs and mRNAs along the WSSV genome from cephalothoraxes of *F. chinensis* during acute WSSV infection. (**A**) Coding structures of the WSSV genome. Arrows represent annotated coding sequences from NCBI. (**B**) Coverage of the mRNA reads (orientation unknown) on a logarithmic scale. (**C**) Coverage of sRNA reads on a logarithmic scale. Bars with a coverage less than 0 represent sRNAs in the reverse direction. Red arrows between (**B**,**C**) indicate regions where “coldspot” of mRNA and sRNA match each other.

**Figure 3:**
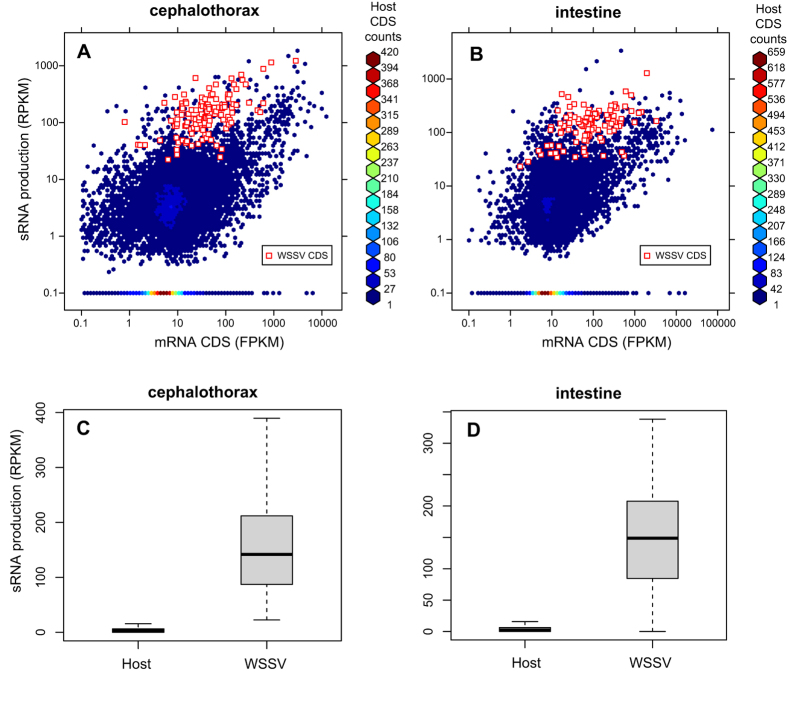
Comparison of sRNA production from coding sequences (CDSs) of WSSV and host genes in the cephalothoraxes and intestines of *F. chinensis* during acute infection. For each CDS, the production of sRNAs has been normalized by the length of the CDS. (**A,B**) Scatter plots in logarithmic style. (**C,D**) Box plots. The sRNA production in the WSSV CDSs (median = 141.9 RPKM) was 53.2 times higher than that in the host CDSs (2.67 RPKM). The difference were very significant (*t*-test, P = 0; *F*-test, P = 0).

**Figure 4:**
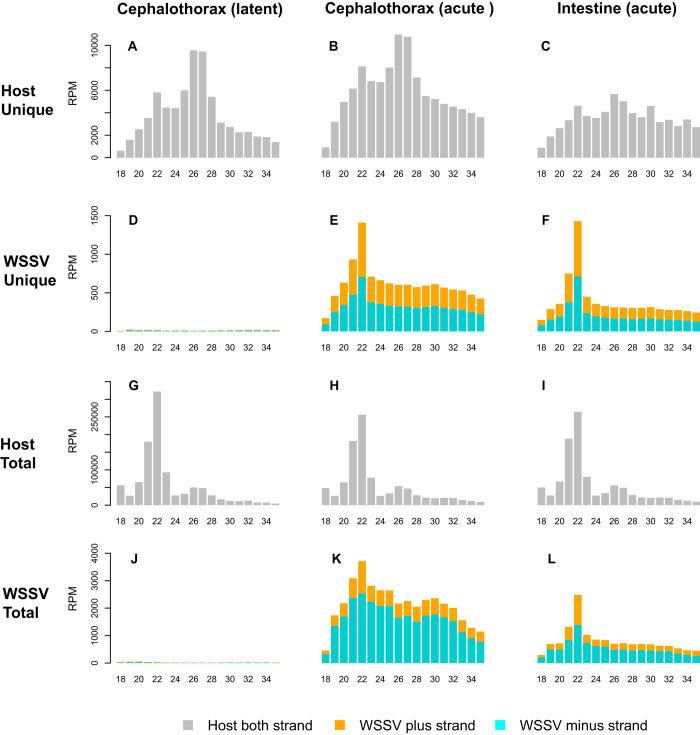
Length distribution of WSSV and host sRNA reads. “Total” and “unique” are abbreviations for total reads and unique reads, respectively.

**Figure 5:**
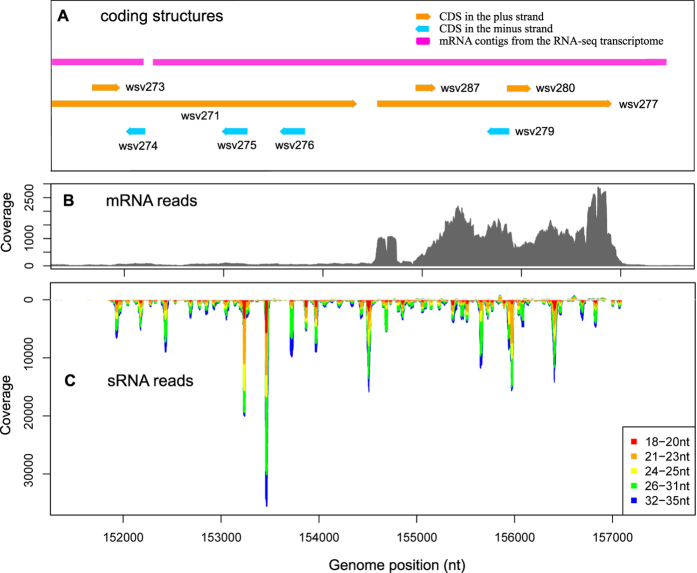
Distribution of sRNAs and mRNAs at the hotspot at ~155 kb in the WSSV genome in cephalothoraxes of *F. chinensis* during acute infection. (**A**) Coding structures. Arrows represent coding sequence annotation from NCBI. (**B**) Sequencing coverage of mRNA reads (orientation unknown). (**C**) Sequencing coverage of sRNA reads. Bars with a coverage of less than 0 represent sRNAs in the reverse direction. The lengths of the sRNAs are indicated in different colors.

**Figure 6:**
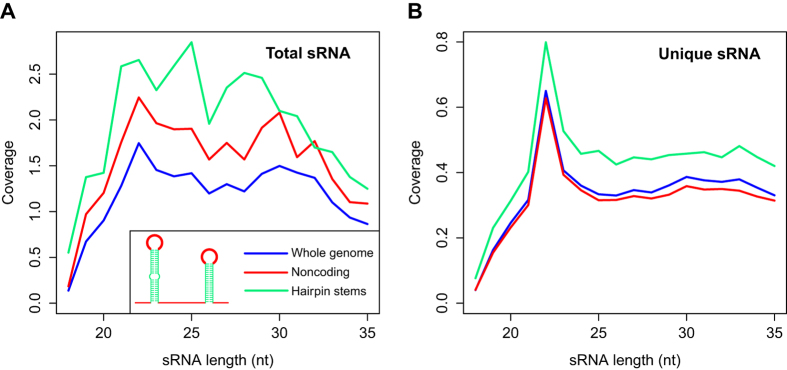
sRNA (18-24 nt) expression in different regions of the WSSV genome during acute infection in the cephalothoraxes of *F. chinensis*. (**A**) Total sRNA coverage. (**B**) Unique sRNA coverage. An illustration of hairpin structures is shown in the legend, with different regions illustrated in the same colors used in (**A,B**). Total sRNA frequency was significantly higher in hairpin structures than in other regions (Wilcoxon rank-sum test, W = 1.0 × 10^10^, P = 2.2 × 10^−16^).

**Figure 7:**
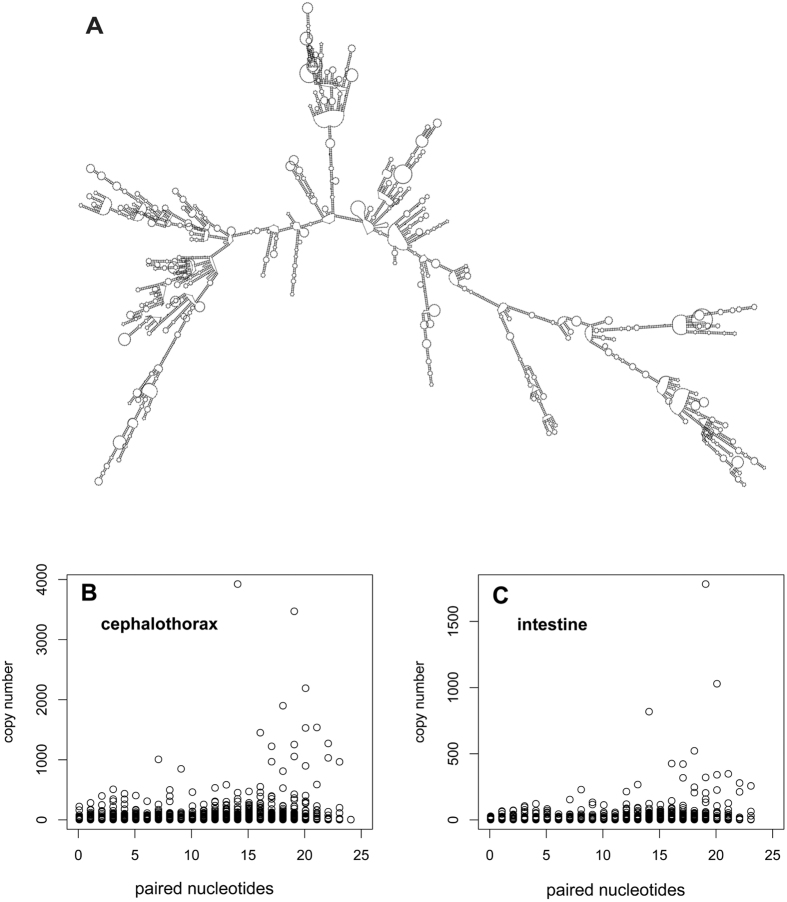
Relationship between sRNA copy numbers and RNA structures at the sRNA hotspot at ~155 kb of WSSV genome during acute infection. (**A**) Predicted RNA structures. (**B,C**) Relationship between the total counts of an sRNA and the number of paired nucleotides corresponding to the sRNA. Unique sRNAs (18–24 nt) were mapped to the RNA secondary structure in (**A**). The mapped site for each individual sRNA was then counted for the paired nucleotides. Nucleotides forming double-stranded bonds were counted, while those in bulge structures were excluded. Using this strategy, the number of paired nucleotides for an sRNA was obtained. This number was related to the copy number of the same unique sRNA, which was obtained by sequencing. Significant differences were found for the copy numbers between sRNAs (average count = 12.0) with higher (≥12) paired nucleotides and those (average count = 7.5) with lower (≤11) paired nucleotides (t = 1.2 × 10^−9^, *t*-test with unequal sample sizes and unequal variances; W = 1.1 × 10^9^, P = 1.6 × 10^−5^, Wilcoxon rank-sum test).

**Figure 8:**
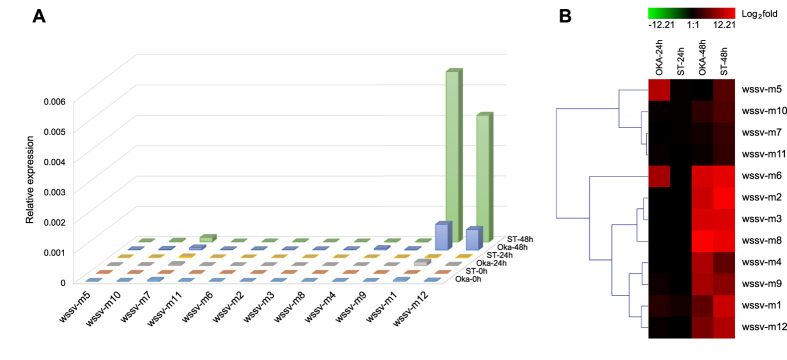
WSSV miRNA expression at 24 and 48 h post-injection in the stomach and the lymphoid organ of *F. chinensis*. **(A)** The viral miRNA expression levels compared with 18S rRNA. **(B)** Fold change of the miRNAs’ expression levels compared with their own levels at hour 0. “OKA” and “ST” stands for lymphoid organ and stomach, respectively.

The flow chart outlines the applied bioinformatic analysis technique (Fig. 1).

### Preparation of shrimp and RNA samples

Cephalothorax samples of WSSV-carrying *F. chinensis* collected during the LI and AI stages of infection, prepared as described in our previous transcriptome study^34^, were used for the transcriptome and sRNA sequencing. Briefly, WSSV-carrying shrimp obtained from a local farm were cultured in tanks, and individuals in the LI and AI stages were classified according to WSSV copy numbers^55^. The cephalothoraxes of 10 LI shrimps and another 10 AI individuals were sampled, frozen, and preserved in liquid nitrogen until DNA or RNA extraction. An independent RNA sample was also obtained from the intestines of AI *F. chinensis* collected at another local farm at a different time, following the same procedures described above.

The shrimp used for the time series and organ-specific miRNA RT-PCR have been described in our previous study^55^. In short, *F. chinensis* received an intramuscular injection of 800 copies of WSSV, and tissues were dissected at different hours post injection (hpi) and immediately preserved individually in liquid nitrogen. Stomachs and lymphoid (Oka) organs from 3 shrimp at 0, 24 and 48 hpi were pooled for RNA extraction.

Total RNA was extracted using Unizol reagent (Sangon, China), treated with DNase I, verified by electrophoresis in a 1% agarose gel and quantified with a NanoDrop 1000 spectrophotometer (NanoDrop, USA).

### Transcriptome sequencing

Transcriptome sequencing was performed as previously described^34^. Briefly, Polyadenylated (polyA+) RNA was purified using Sera-mag oligo (dT) beads, fragmented, reverse-transcribed using random hexamers, end-repaired, and adaptor-ligated. Products of 300–500 bp were separated, amplified, and pair-end sequenced using a Genome Analyzer II (Illumina, San Diego, USA) at the Beijing Genomics Institute (BGI), Shenzhen, China.

### sRNA sequencing

Ten micrograms of total RNA was used to construct the sRNA libraries with the Small RNA Sample Prep Kit (Illumina, San Diego, USA). Briefly, sRNA fragments with an approximate length of 18–35 nt were isolated and purified following 15% denaturing polyacrylamide gel electrophoresis (PAGE). Subsequently, 3′ and 5′ RNA adaptors were ligated to the sRNAs using T4 RNA ligase. RT-PCR was performed to reverse transcribe the adaptor-ligated sRNAs into cDNAs, which were further amplified for 15 PCR cycles. The products were purified using 10% PAGE and checked for size, purity, and concentration with an Agilent 2100 Bioanalyzer (Agilent, Santa Clara, USA). The sRNA libraries were sequenced using an Illumina/Solexa 1G Genome Analyzer (Illumina, San Diego, USA) at the BGI. The LI and AI sRNA sequencing data have been submitted to the SRA database at the NCBI and are now available under accession numbers SRX807107 and SRX806880, respectively.

### Classification of host sRNAs

The clean sRNA reads were annotated by comparison with the Rfam non-coding RNA database (http://xfam.org/) and the miRBase miRNA database (http://mirbase.org/), using Blastn. The majority of total sRNAs were successfully classified into known sRNA categories, which made up 67%, 60% and 70% of the total sRNAs from the LI cephalothorax, AI cephalothorax, and AI intestine, respectively (Supplementary Fig. S1).

### Identification of virus-derived sRNAs

Viral sRNAs were identified by comparison with WSSV genome and transcriptome sequences.

Four WSSV genomes were downloaded from NCBI (GI: 19481591, 426202315, 417410589 and 58866698). After removing adaptor or low-quality sequences, sRNAs with a length of 18–35 nt were mapped to the WSSV genome using bowtie2 (http://bowtie-bio.sourceforge.net) and SAMtools (http://samtools.sourceforge.net/) to separate WSSV and host sRNAs. Because the mapping results were very similar between the 4 versions, the most recently updated genome, GI417410589 was chosen for subsequent analyses. The genomic distribution of mapped vsiRNAs was visualized using R (https://www.r-project.org/).

Transcriptome sequences from the same sample were annotated as previously described. Transcripts matching plants or microbes other than WSSV were excluded from the analysis. Ribosomal RNA transcripts were also removed. sRNAs were also mapped to annotated transcripts using bowtie2 and SAMtools. The sRNA expression of each transcript was calculated as RPKM and then integrated according to the NR annotation to summarize the expression of transcripts from the same gene.

### Comparison of sRNA production from host and WSSV CDSs

To evaluate whether the unclassified short RNAs (Supplementary Fig. S1) were degradation products, they were mapped to protein coding sequences (CDSs) from the transcriptome sequencing data obtained from the same samples^34^. The CDSs were obtained by aligning mRNAs to known proteins in the NCBI NR database, using Blastx. For AI cephalothorax, 120 WSSV CDSs and 17,240 host CDSs (FPKM ≥ 0.1) were compared for unique sRNAs mapping to the sense strand of each CDS. A considerable proportion (27.6%) of the host CDSs showed no sRNA production; by contrast, 100% of the WSSV CDSs displayed sRNA production. sRNA distribution frequency in the CDSs was calculated in RPKM. Difference in sRNA frequencies between host CDSs and WSSV CDSs were compared using *t*-test and *F*-test.

### Genomic characterization of virus-derived sRNAs

We refer to the viral strand that is transcribed from left to right as the plus strand and to its complement as the minus strand.

At first, the viral sRNAs were evaluated for miRNA characteristics. Sequences from the coding and non-coding regions of the WSSV genome were obtained according to the gene structure annotation (GI417410589, NCBI), using in-house Perl (https://www.perl.org/) scripts. pre-miRNA-like hairpin structures along the non-coding region of the WSSV genome were predicted with the srnaloop algorithm (http://arep.med.harvard.edu/miRNA/). The stability of the structures was evaluated with RNAfold (http://www.tbi.univie.ac.at/RNA/) based on calculations of minimum free energy (MFE < −25 kcal/mole). Then the sequences of both arms and the loop regions of the structures were retrieved with Perl scripts. The sRNA frequencies in the hairpin arms, loops, and other genomic regions were compared, for which the regions were trimmed by 24 nt to exclude sRNAs that stretched across the borders. For sRNAs in the range of 18–24 nt (the length of miRNAs), the differences were checked using the Wilcoxon rank-sum test and *t*-test, using R.

In order to assess the viral sRNAs for piRNA characteristics, the nucleotide preferences of sRNAs and the distance between the 5′ ends of the sense and antisense sRNAs were visualized using R.

To verify the association between the sRNA frequency and RNA secondary structures in long sequence ranges, the RNA secondary structures of the sRNA hotspot at 151–157.5 kb in the minus strand of the WSSV genome were predicted with RNAfold (MFE < −25 kcal/mole).

### Classification of viral siRNA and miRNA

Stacks of multiple offset sRNA reads (21–23 nt) covering a continuous region were classified as possible siRNAs^33^ if the difference in their 5′ start positions was >5 nt. Next, two strategies, the miRDeep2 and the srnaloop methods, were combined to identify viral miRNAs among the remaining sRNAs. Initially, the miRDeep2 pipeline analysis was performed according to Friedlander *et al*.^56^. Briefly, all of the clean reads were mapped to the genomes, and potential pre-miRNA structures mapped by stacking 18–24-nt sRNAs were excised from the genome and assessed in terms of their energetic stability by RNAfold to identify pre-miRNA hairpins. If a read fell within a pre-miRNA-like structure resembling a pair of Dicer-processed miRNAs and its miRNA-star, a score was given to reflect the likelihood of the pre-miRNA being genuine. To recover miRNAs that might have been overlooked by miRDeep2, another procedure was also adopted. This procedure was initiated by filtering for pre-miRNA-like structures along the non-coding region of the WSSV genome using the srnaloop algorithm and by retrieving sequences in both hairpin arms. A stack of 18–24 nt sRNA reads (copy number ≥2) residing in a hairpin arm was considered a possible miRNA if there were no adjacent overlapping sRNA stacks. Repetitive sequences were removed from the miRNA candidates. To distinguish novel miRNAs from known miRNAs, the predicted miRNA was compared with all registered miRNAs in miRBase and with previously reported WSSV miRNAs that were not registered in miRBase^30^,^57^. Finally, the remaining 21–23-nt sRNAs that aligned with the WSSV genome were considered to be potential viral siRNAs.

### Quantitative real-time PCR

Mature miRNAs were first reverse-transcribed with a looped RT primer^41^ (Supplementary Dataset S1, LC-Sciences, Houston, USA) complementary to the 3′ end of the miRNA, and the subsequent cDNA was quantified by real-time PCR using Platinum SYBR Green qPCR SuperMix (Thermo Fisher, Waltham, USA) with the ABI PRISM® 7900HT system (Thermo Fisher, Waltham, USA)^41^. The expression levels were normalized to 18S rRNA and evaluated using the comparative CT method. Statistical analyses were performed using paired Student’s *t*-tests.

### Viral miRNA target analysis

Unlike plant miRNAs, which bind to the coding regions of their target mRNAs, animal miRNA binding sites are usually confined to the 3′ UTR^58^. Binding sites in 3′ UTRs have been reported to show high regulatory efficacy, while those in ORFs and 5′ UTRs have only marginal or no efficacy^58^. Thus, we focused on 3′ UTRs for miRNA target prediction. Two strategies were used to predict WSSV miRNA targets, namely the miRanda strategy^42^ and the Targetscan strategy^58^. The miRanda strategy was used to assess the potential scope of the influence of viral miRNAs on host genes, whereas the Targetscan strategy was performed to identify functionally important target genes that are conserved among different host species or virus strains.

For the miRanda strategy, mRNA data obtained from the same samples as the miRNA data were used as the target background, as described previously^34^. The coding structures of the mRNA transcripts were analyzed using a combination of 2 algorithms. First, the transcripts were compared to the sequences deposited in the NCBI protein database (Nr) to identify orthologous coding sequences, using Blastx (E = 1e−5). Second, for transcripts with no known homology, ESTscan (http://estscan.sourceforge.net/) was used to predict the coding structure. The 3′ UTR sequences were retrieved and evaluated for miRNA binding sites using miRanda^42^, which requires strict alignment in the seed region (miRNA offset positions 2–8).

For the Targetscan strategy, the 3′ UTR data from three host species and four WSSV strains were used to predict conserved target genes. For the host genes, in addition to the *F. chinensis* data, the transcriptome sequences of *Litopenaeus vannamei* and *Exopalaemon carincauda* assembled in our previous study were used^59^,^60^. Homologous transcripts were identified by blast alignment (E = 1e−5) between unigenes of the three hosts, requiring bi-directional best hit (BBH). Coding structures were analyzed for 46,813, 66,815, and 68,693 transcriptome unigenes from *F. chinensis*, *L. vannamei*, and *E. carincauda*, resulting in 6,257, 6,031, and 9,322 3′ UTRs, respectively. Homologous 3′ UTR sequences were identified in 602 genes, and were searched for miRNA targets. For viral genes, homologous 3′ UTRs were extracted from four WSSV genomes in the NCBI database (GI: 19481591, 417410589, 426202315, and 58866698). mRNAs without NCBI (Nr) annotation were excluded. 3′ UTRs longer than 25 nt were retrieved and aligned using MUSCLE (http://www.drive5.com/muscle/). Conserved 3′ UTR regions were searched for miRNA binding sites using Targetscan^58^.

### Visualization of the viral miRNA regulation network

A viral miRNA regulatory network containing 12 WSSV miRNAs and their target genes was constructed. The expression levels and fold-changes of the miRNAs and their target genes during AI in shrimp cephalothoraxes were integrated into the network, which was visualized with Cytoscape (http://cytoscape.org/). The miRNAs and their target genes are represented by different symbols. Fold changes in the expression levels of miRNAs and targets during acute WSSV infection are indicated by the color gradient. The absolute expression level of an miRNA (RPKM) or a target gene (FPKM, fragments per kilobase of transcript per million mapped reads) is indicated by the size of the symbol.

## Additional Information

**How to cite this article**: Liu, C. *et al*. Virus-derived small RNAs in the penaeid shrimp *Fenneropenaeus chinensis* during acute infection of the DNA virus WSSV. *Sci. Rep.* **6**, 28678; doi: 10.1038/srep28678 (2016).

## Supplementary Material

Supplementary Information

Supplementary Dataset S1

Supplementary Dataset S2

Supplementary Dataset S3

## Figures and Tables

**Table 1:** WSSV-derived sRNA expression in the cephalothoraxes of *F. chinensis* at LI and AI stages.

Category	Stages	Parameter	21–23 nt^a^	18–35 nt
Unique sRNA	Latent	Count	204	1,664
		Density^b^	0.00033	0.0027
		21–23 nt/18–35 nt	0.12	
	Acute	Count	34,904	131,637
		Density	0.06	0.22
		21–23 nt/18–35 nt	0.27	
	Acute/latent	Fold	171	79
Total sRNA	Latent	RPM^c^	23	194
		21–23 nt/18–35 nt	0.12	
	Acute	RPM	8719	36631
		21–23 nt/18–35 nt	0.24	
	Acute/latent	Fold	385	188

^a^“21–23nt” represents sRNAs in the size range of 21–23 nucleotides, which is the typical length of siRNA. “18–35 nt” stands for sRNAs in the range of 18–35 nucleotides, which covers the length ranges of siRNA, miRNA and piRNA.

^b^The strand-specific density here was calculated as the number of unique WSSV-derived sRNAs divided by twice the length of the WSSV genome.

^c^RPM: WSSV-derived sRNA reads per million sRNA reads.

**Table 2:** WSSV miRNAs verified by RT-PCR.

ID	Sequence	Genome Position			Pri-miRNA ID^a^
		Strand	Position	5′/3′	
wssv-m1	GUUGUUGUUCAUGUUGAGGGCAUU	−	152840~152863	5′	33576
wssv-m2	AGAAGAGGACUUUGGGGUAGAC	−	79223~79244	5′	31474
wssv-m3	UGACGAUAAGAGGAGGCAGUAG	+	236406~236427	5′	33547
wssv-m4	GAAAUGGCUGUCCAGAAAUCUGG	+	272861~273501 273826~272883 273523~273848	5′	/
wssv-m5	CAGGUCUCUUCUAGGUAGAAG	−	41679~41699	5′	39470
wssv-m6	CUGGAGAGACUAUAGGGGAAUA	+	173945~173966	3′	33544
wssv-m7	GAAAGGUUGUUGAUGGAGAUG	−	290022~290042	3′	33511
wssv-m8	GCGUCUCUUGAGUCUUGUAGUA	−	250561~250582	5′	37970
wssv-m9	UAAGAGAAUAAGGAGAAUCUGAG	+	246949~246971	3′	39453
wssv-m10	GUGACUGGAGUGUUGAUUGGGU	+	248192~248213	5′	/
wssv-m11	UAACAUUAUCGUGGAACAGAAA	−	43233~43254	3′	39471
wssv-m12	GGGGUUUCGUUUGUGGUUG	−	154722~154740	5′	33576

^a^Transcriptome sequences from Li *et al*.^34^. The prefix “Unigene” was omitted. The sequences can be found in Text S3 in the Supplemental material.
