# Supplementary material for: Virus-derived small RNAs in the penaeid shrimp Fenneropenaeus chinensis during acute infection of the DNA virus WSSV
Source: Sci Rep. 2016 Jun 28;6:28678. doi: 10.1038/srep28678 (PMC4923869; doi:10.1038/srep28678)
Supplement: Supplementary Information [file srep28678-s1.pdf]

Virus-derived small RNAs in the penaeid shrimp *Fenneropenaeus chinensis* during acute infection of the DNA virus WSSV

Chengzhang Liu[1], Fuhua Li[1,2], Yumiao Sun[1], Xiaojun Zhang[1,2],

Jianbo Yuan[1], Hui Yang[1], Jianhai Xiang[1,2,*]

[1]Key Laboratory of Experimental Marine Biology, Institute of Oceanology,

Chinese Academy of Sciences, Qingdao 266071, China

[2]Laboratory for Marine Biology and Biotechnology, Qingdao National Laboratory

for Marine Science and Technology, Qingdao 266071, China

*Address correspondence to Jianhai Xiang, jhxiang@qdio.ac.cn

Table S1 WSSV genes potentially regulated by WSSV miRNAs

**Target genes**	**WSSV miRNAs**	**Samples[a]**
ORF126	N1,m1	CEP+INT
ORF14	m11	CEP+INT
ORF61	N1,m1,m7	CEP+INT
putative DNA polymerase	m2,m6	CEP+INT
WSSV156	m11	CEP
WSSV507	m6	CEP+INT
wsv026	m1,m2	CEP+INT
wsv067	m6	CEP
wsv133	m1,m6	CEP+INT
wsv139	m12,m2	CEP+INT
wsv151	N1,m1,m2,m6,m9,m10	CEP+INT
wsv184	N1,m7	CEP+INT
wsv206	m1	CEP+INT
wsv269	miR-211	CEP
wsv277	m1,m11	CEP+INT
wsv282	m1,m6	CEP+INT
wsv303	m7,m9	CEP+INT
wsv340	N1,m6	CEP+INT
wsv387	m7	CEP+INT
wsv395	m1,m12,miR-211	CEP+INT
wsv415	m1,m7,m12	CEP+INT
wsv442	m1	CEP

[a]**CEP:** cephalothoraxes; **INT**: intestine

Table S2 Virus-derived sRNA profiles of animal viruses

Virus	Genome	Host	Genome covered[a]	Length (nt)	Strand polarity[b]	5' bias[c]	Class	Reference[d]
ANV	+ssRNA	fruit fly	/	27~28	+	/	piRNA	(Wu et al., 2010)
DBV	dsRNA	fruit fly	/	~21	+	U	siRNA	(Wu et al., 2010)
DCV	+ssRNA	fruit fly	/	~21	+	/	siRNA	(Wu et al., 2010)
DCV	+ssRNA	fruit fly	/	27~28	+	/	piRNA	(Wu et al., 2010)
DCV	+ssRNA	fruit fly	/	~21	+87.4%	/	siRNA	(Sabin et al., 2013)
DENV2	+ssRNA	mosquito	/	20~23	+/-	/	siRNA	(Scott et al., 2010)
DENV2	+ssRNA	mosquito	/	24~30	+	/	piRNA	(Scott et al., 2010)
rDENV-4	+ssRNA	mosquito	/	~21	+59.3%	/	/	(Schirtzinger et al., 2015)
rDENV-4	+ssRNA	primate	/	~24	+99.0%	U	/	(Schirtzinger et al., 2015)
DNV	+ssDNA	insects	78.5%	~21	+87.5%	/	siRNA	(Ma et al., 2011)
DTrV	+ssRNA	fruit fly	/	~21	+	/	siRNA	(Wu et al., 2010)
DTV	dsRNA	fruit fly	/	/	/	/	siRNA	(Wu et al., 2010)
DXV	dsRNA	fruit fly	78~91%	~21	+	+U	siRNA	(Wu et al., 2010)
FHV	+ssRNA	fruit fly	/	~21	~50%	/	siRNA	(Aliyari et al., 2008; Flynt et al., 2009; Han et al., 2011)
FHV	+ssRNA	nematode	93%	23	~50%	U	siRNA	(Wu et al., 2010)
HaSNPV	dsDNA	cotton bollworm	/	~20	/	/	siRNA	(Jayachandran et al., 2012)
HiPV	+ssRNA	planthopper	72.9%	21~22	+67.4%	U	siRNA	(Xu et al., 2014)
HoCV-1	+ssRNA	leafhopper	100%	~21	+95%	/	siRNA	(Nandety et al., 2013)
HoVRV	dsRNA	leafhopper	100%	~21	-	/	siRNA	(Nandety et al., 2013)
IIV-6	dsDNA	insects	/	~21	+47%	/	siRNA	(Bronkhorst et al., 2012)
LGTV	+ssRNA	tick	/	~22	~50%	A	siRNA	(Schnettler et al., 2014)
MNV	+ssRNA	mosquito	/	/	/	/	/	(Wu et al., 2010)

RSV	-ssRNA	plant/insect	/	20-24	~50%	U	siRNA	(Xu et al., 2012)
RVFV	-ssRNA	insect/vertebrates	/	24~28	-90%	U	piRNA	(Leger et al., 2013)
RVFV	-ssRNA	insect/vertebrates	/	~21	~50%	/	siR/miR	(Leger et al., 2013; Sabin et al., 2013)
SFV	+ssRNA	mosquito	/	~21	+	/	siRNA	(Siu et al., 2011)
SINV	+ssRNA	mosquito	99%	~21	+	/	siRNA	(Myles et al., 2008; Wu et al., 2010)
TBEV	+ssRNA	tick	/	~22	~50%	/	siRNA	(Schnettler et al., 2014)
VACV	dsDNA	insect/mammal	most	~21	+68.8%	/	siRNA	(Sabin et al., 2013)
VSV	-ssRNA	insect/vertebrates	/	~21	+44/53%	/	siRNA	(Mueller et al., 2010)
VSV	-ssRNA	insect/vertebrates	/	~21	+54.5%	/	siR/miR	(Sabin et al., 2013)
WNV	+ssRNA	fruit fly	/	~25	/	/	siRNA	(Chotkowski et al., 2008)
WNV	+ssRNA	mosquito	82~92%	~21	vary	/	siRNA	(Brackney et al., 2009)
WSSV	dsDNA	crustacean	/	~22	/	/	miRNA/siRNA	(He and Zhang, 2012; Huang et al., 2014; Huang and Zhang, 2013)

[a] Proportion of viral genome length covered by sRNA reads.

[b] Polarity of viral sRNA: "+" and "–" stand for sRNAs matching the positive and negative strands of virus genome, respectively. Numbers in percentage represent proportion of sRNAs matching the corresponding strand of virus genome.

[c] Nucleotide preference at 5' end of viral sRNA.

[d] Please find the references at the end of the Supplemental Materials.

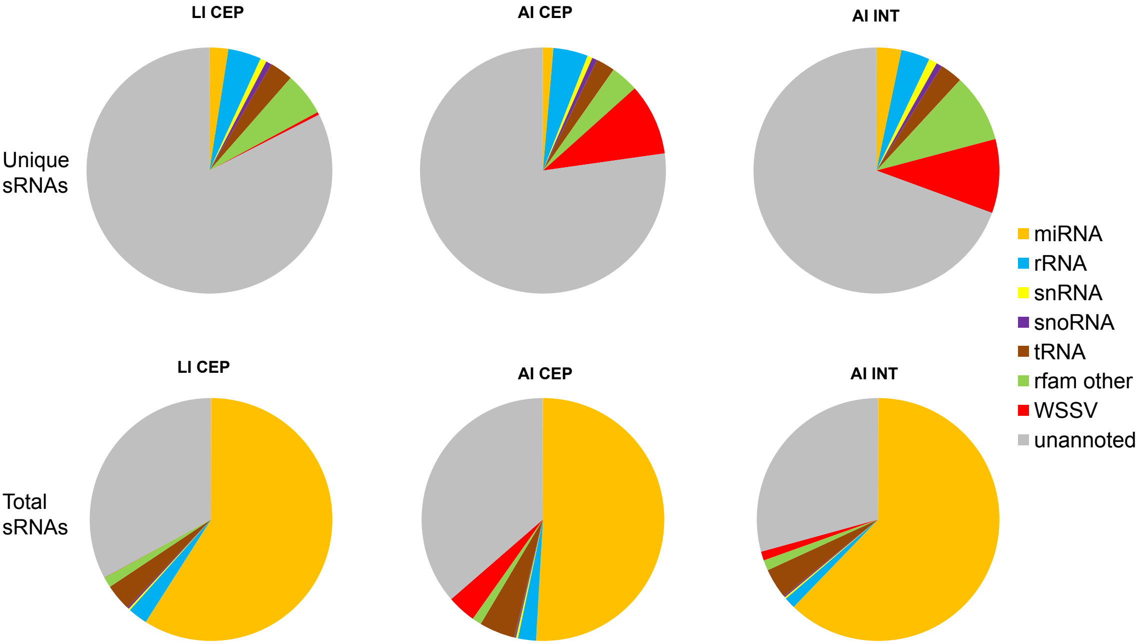

FIG S1 Classification of sRNA sequencing reads. The clean reads (17-35nt) were annotated in a preferential order by comparing to the WSSV genome, the Rfam non-coding RNA database and miRBase miRNA database. CEP and INT stand for cephalothoraxes and intestine of *F. chinensis*, respectively. LI and AI represent latent or acute stages of infection.

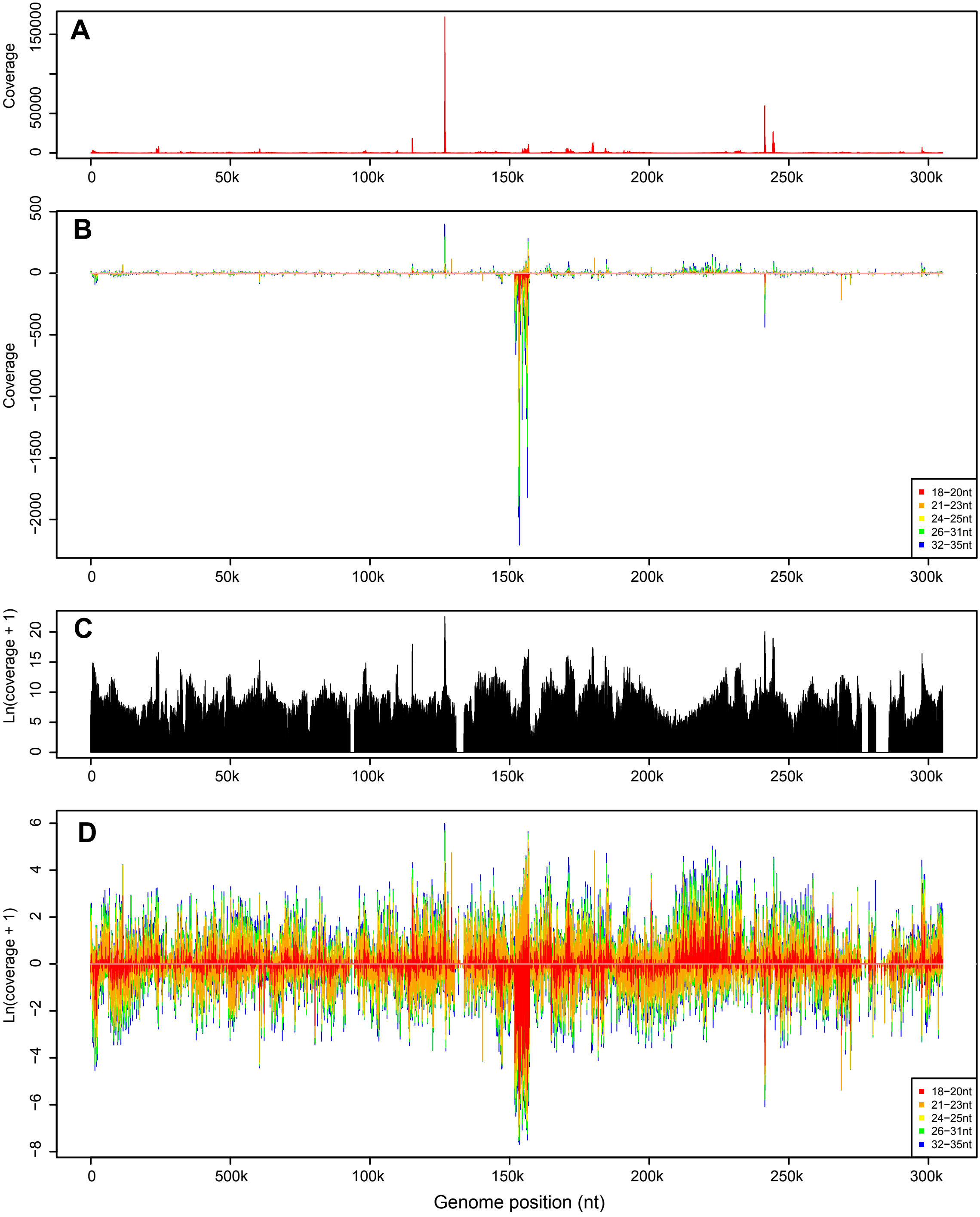

FIG S2 Expression of sRNAs and mRNAs along WSSV genome in intesine of *F. chinensis* during acute infection. **(A)** Sequencing coverage of mRNA reads (orientation unknown). **(B)** Sequencing coverage of sRNA reads. Bars beneath coverage 0 represent sRNAs in the reverse direction of genome. The lengths of sRNAs are indicated with different colors. **(C)** Coverage of mRNA reads in logarithmic scale. **(D)** Coverage of sRNA reads in logarithmic scale. Bars beneath coverage 0 represent sRNAs in the reverse direction.

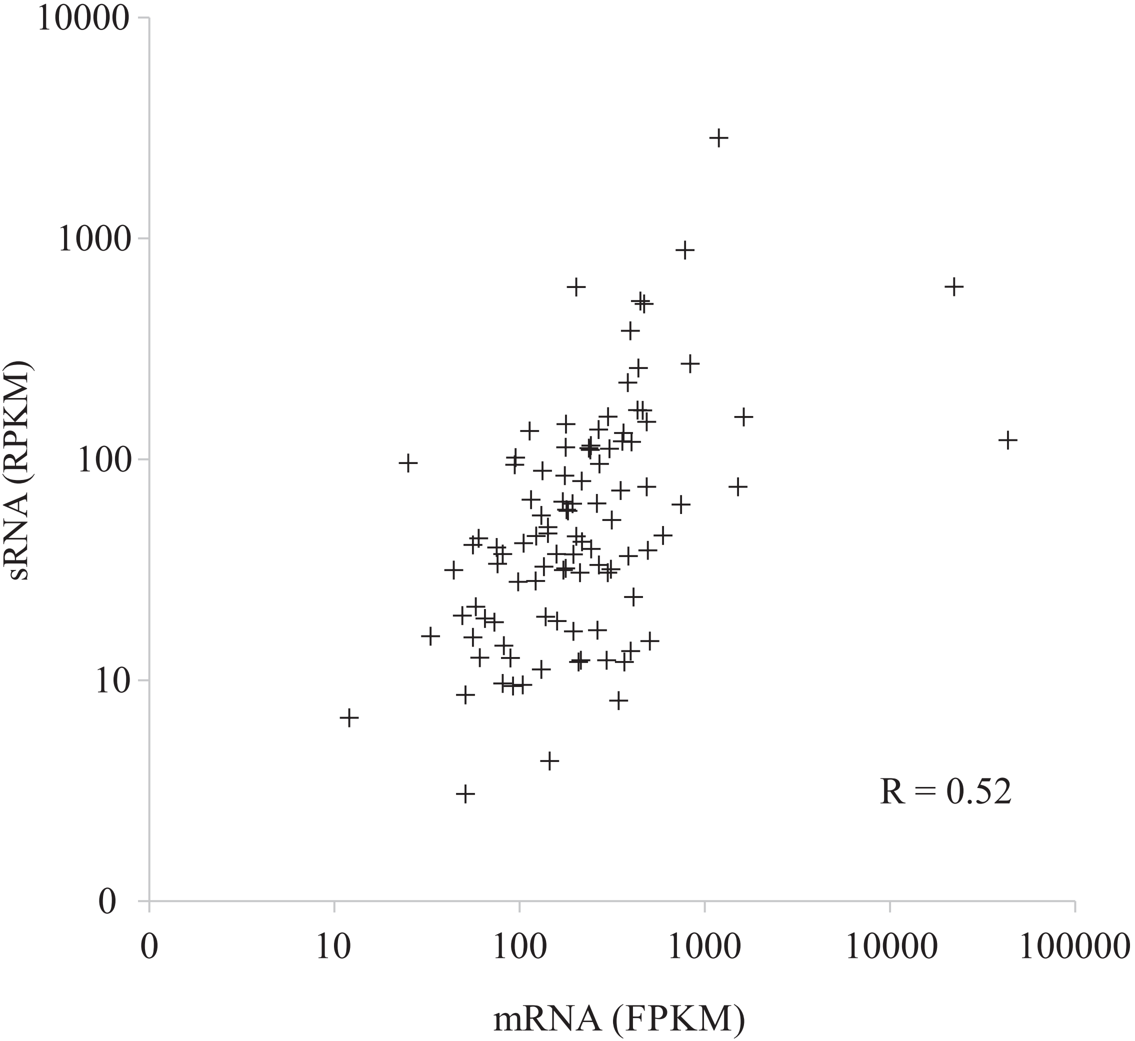

FIG S3 Relations between the expressions of sRNA and mRNA of WSSV in cephalothoraxes of *F. chinensis* during acute infection.

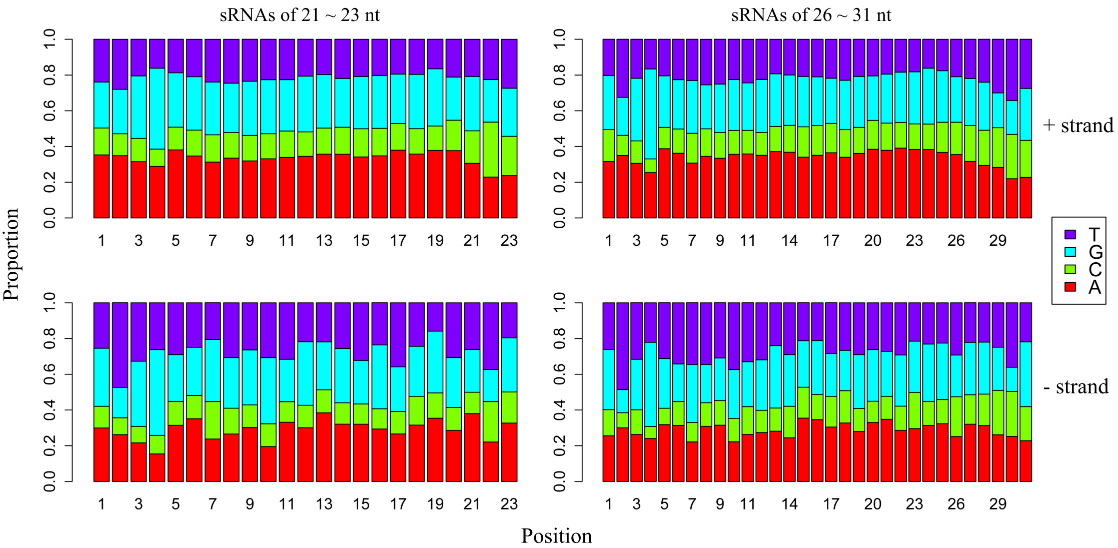

FIG S4 WSSV sRNAs did not show nucleotide preference of canonical piRNA in shrimp cephalothoraxes during acute infection. Nucleotide frequencies of total sRNAs at each position are shown with bars in different colors. Figures in the upper row show sRNAs mapped to the WSSV genome in the 5’-3’ direction, the lower two figures show sRNAs matched to the reverse complement direction of the genome. Figures in the left column show sRNAs of length 21~23 nt, which is most aboundant in the AI sample. Figures in the right column show sRNAs of length 26-31 nt, which is in the range of canonical piRNA.

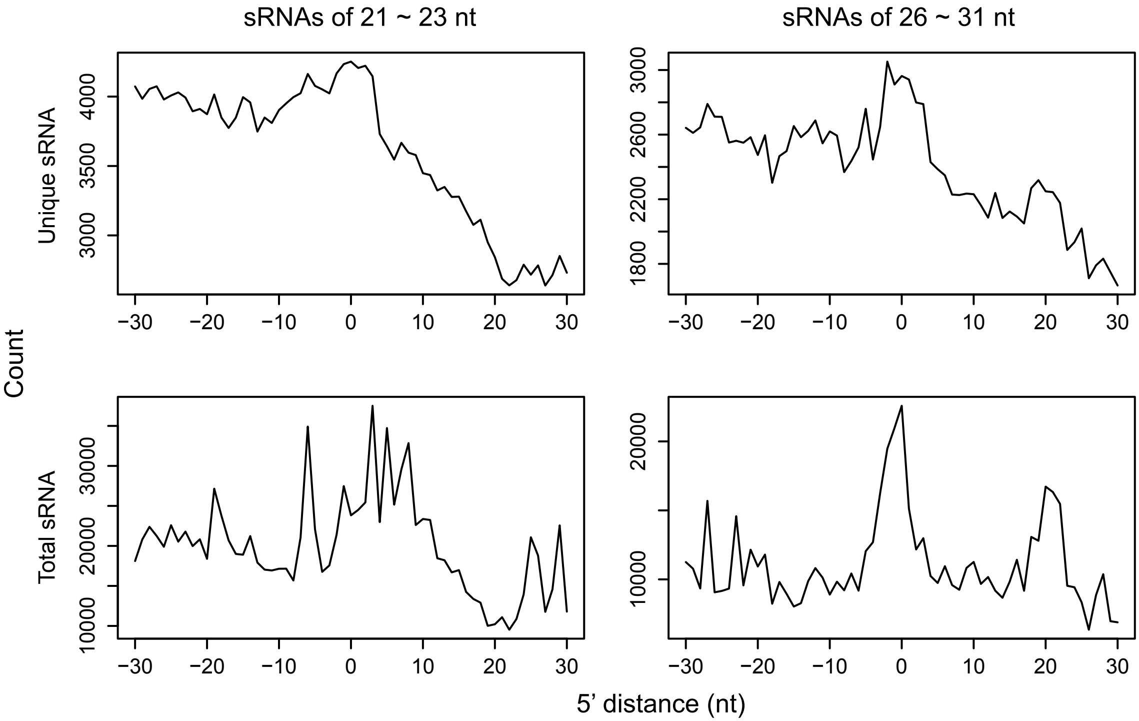

FIG S5 WSSV sRNAs did not show typical 5’ distances (10nt) of canonical piRNA in shrimp cephalothoraxes during acute infection. Frequency map of the distance between sRNAs that mapped to opposite strands of the WSSV genome.

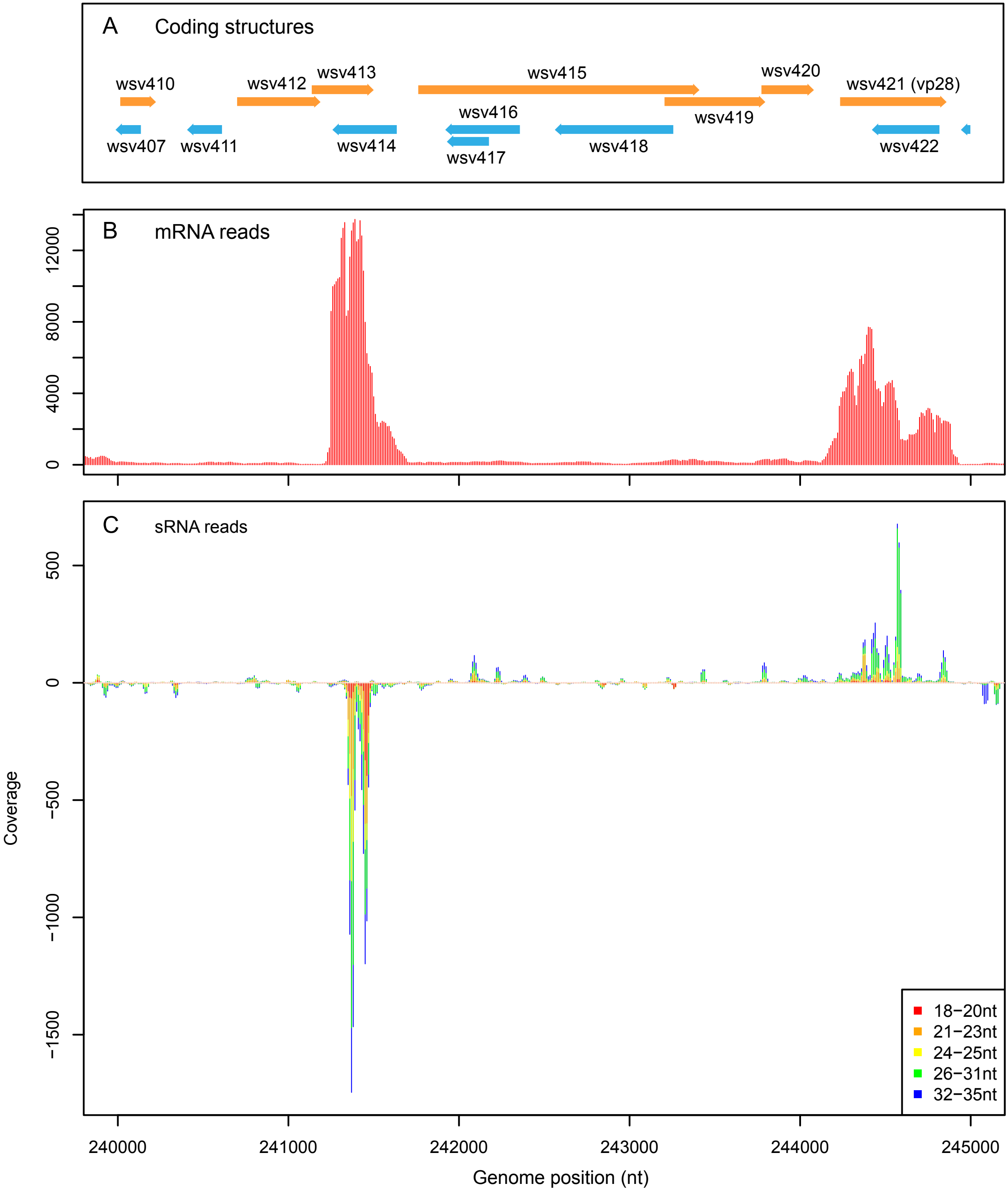

FIG S6 Expression of sRNAs and mRNAs around vp28 gene in WSSV genome during acute infection in shrimp cephalothoraxes. (**A**) Coding structures. Arrows represent coding sequences annotation from NCBI. Orange arrows indicateCDS in plus strand, while cyan arrows indicate CDS in minus strand. (**B**) Sequencing coverageof mRNA reads (orientation unknown). (**C**) Sequencing coverage of sRNA reads. Bars beneath coverage 0 represent sRNAs in the reverse direction. The lengths of sRNAs are indicated with different colors.

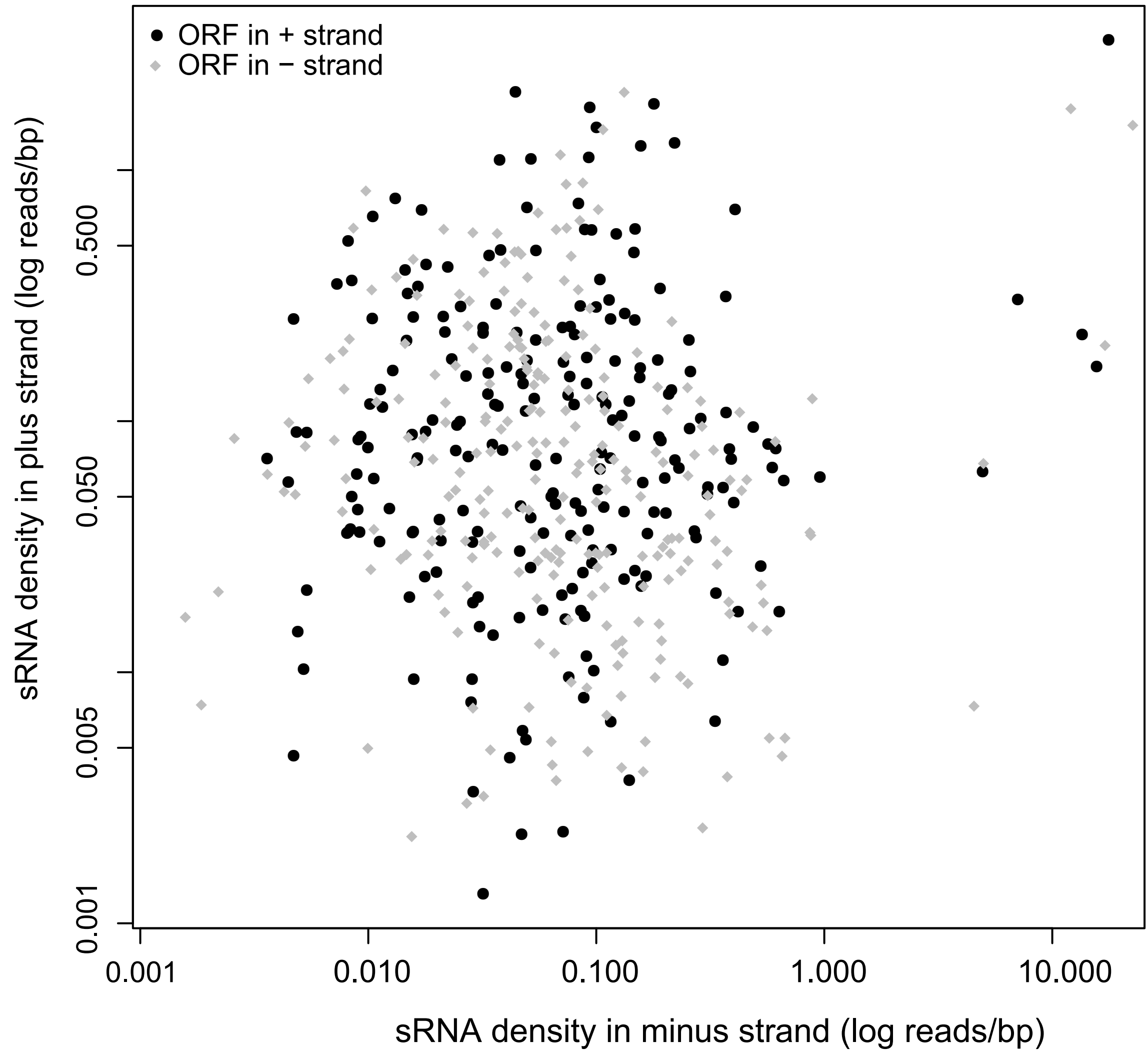

FIG S7 Scatter plot of sRNAs from individual ORFs that map to the WSSV plus and minus strands in shrimp cephalothoraxes during acute infection. For each ORF, the total number of sRNAs derived from each strand was divided by the length of the ORF. The data were log-transformed. Black circles and grey diamonds indicate ORFs on the plus and minus strands of the WSSV genome, respectively.

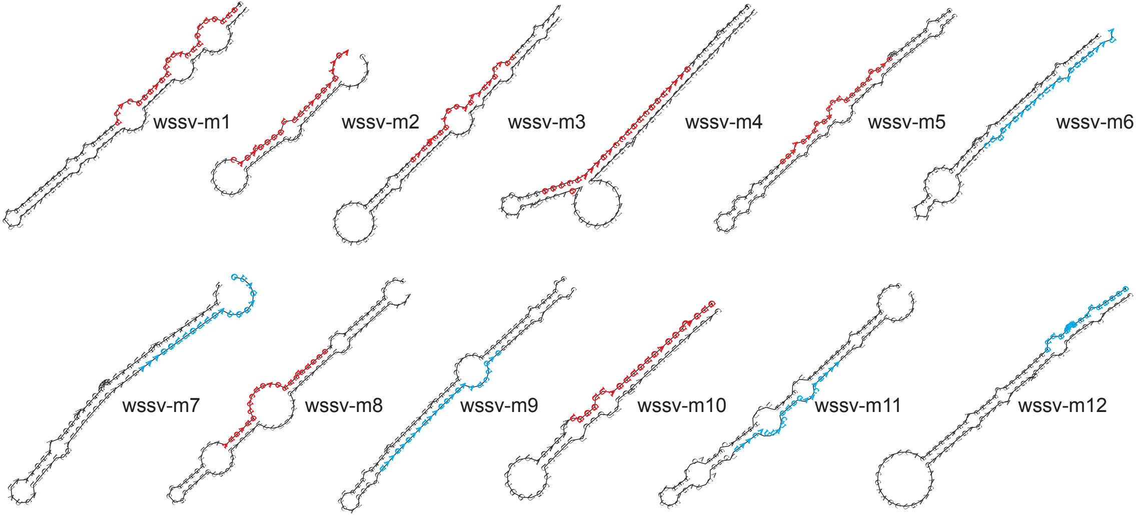

FIG S8 Structures of candidate WSSV miRNA precursors. Pre-miRNA structures were predicted using RNAfold (MFE < -25 kcal/mole). Red color indicates mature miRNAs located at the 5'-arm (5p) of the precursor, and blue indicates mature miRNAs located at the 3' arm (3p).

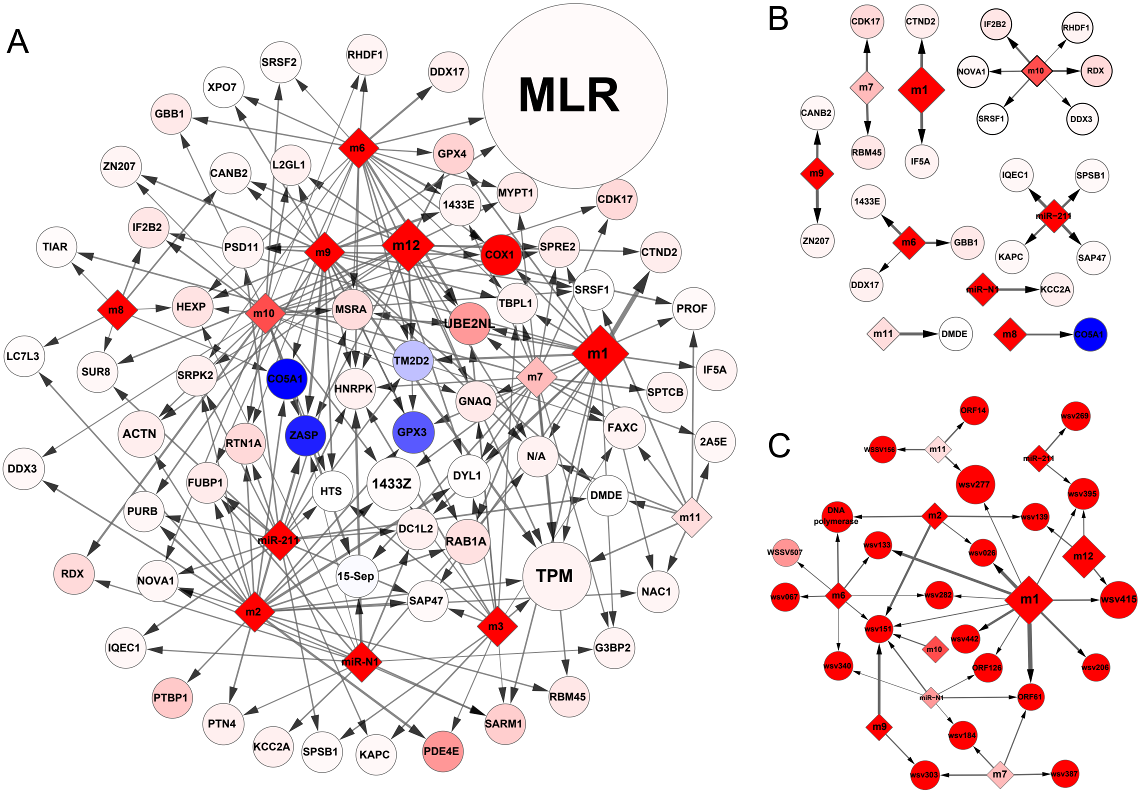

FIG S9 Visualization of viral miRNA regulation network. Diamonds represent miRNAs, while circles represent target genes. Red or blue colors indicate up-regulation or down-regulation during acute infection, respectively. Size of a symbol indicates expression level of a miRNA or target gene. **(A)** Target genes that are shared in three hosts (*F. chinensis, L. vannamei and E. carincauda*). **(B)** Target genes with binding sites that are evolutionally conserved. **(C)** Predicted regulation of WSSV miRNAs on its own mRNAs.

Text S1

Small RNA reads from acute WSSV-infected cephalothorax of *F. chinensis* which overlapped with the vp28 siRNA probes of Huang et al. (2013).

```
>inf_0160452_x3
ACAACACTGTGACCAAGACCATCGAAACCCACA
>inf_0210481_x2
CACAACACTGTGACCAAGACCATCGAAACCCACA
>inf_0317586_x2
CAACACTGTGACCAAGACCATCGAAACCCAC
>inf_0362208_x1
AACACTGTGACCAAGACCATCGAAACCCA
>inf_0364443_x1
AACACTGTGACCAAGACCATCGAAACCCACA
>inf_0747546_x1
CACAACACTGTGACCAAGACCATCGAAACCCA
>inf_0857103_x1
CAACACTGTGACCAAGACCATCGAAACCCACA
>inf_0873829_x1
TGTGACCAAGACCATCGAAACCCACACAGGCA
>inf_1053362_x1
AACACTGTGACCAAGACCATCGAAACCCACACA
>inf_1174596_x1
ACTGTGACCAAGACCATCGAAACCCACA
>inf_1186243_x1
ACAACACTGTGACCAAGACCATCGAAACCCA
>inf_1207632_x1
TGACCAAGACCATCGAAACCCACACAGGC
>inf_1271115_x1
CAACACTGTGACCAAGACCATCGAAACCCACAC
>inf_1576499_x1
AACACTGTGACCAAGACCATCGAAACCCAC
```

Text S2

Sequences of the 12 WSSV pre-miRNAs were conserved in 4 WSSV strains (gi58866698, gi426202315, gi721172032 and gi19481591).

```
MUSCLE (3.8) multiple sequence alignment
```

wssv-m1->gi426202315:152245-152334
GGTTGTTGTTCATGTTGAGGGCATTGTTGTTGTTGTTGTTCGTCTTCGAACGGCATCACC
wssv-m1->gi58866698:91297-91386
GGTTGTTGTTCATGTTGAGGGCATTGTTGTTGTTGTTGTTCGTCTTCGAACGGCATCACC
wssv-m1->gi721172032:147629-147718
GGTTGTTGTTCATGTTGAGGGCATTGTTGTTGTTGTTGTTCGTCTTCGAACGGCATCACC
wssv-m1->gi19481591:120829-120918
GGTTGTTGTTCATGTTGAGGGCATTGTTGTTGTTGTTGTTCGTCTTCGAACGGCATCACC

****

wssv-m1->gi426202315:152245-152334
GACATCATTTCTTCATTCGAGGTAGAGACT
wssv-m1->gi58866698:91297-91386
GACATCATTTCTTCATTCGAGGTAGAGACT
wssv-m1->gi721172032:147629-147718
GACATCATTTCTTCATTCGAGGTAGAGACT
wssv-m1->gi19481591:120829-120918
GACATCATTTCTTCATTCGAGGTAGAGACT
****

MUSCLE (3.8) multiple sequence alignment

wssv-m2->gi58866698:164455-164511
AGAAGAGGACTTTGGGGTAGACATTTTCTTCCTCTCCCCTTCCAGGTCCTTAATAAC
wssv-m2->gi721172032:221267-221323
AGAAGAGGACTTTGGGGTAGACATTTTCTTCCTCTCCCCTTCCAGGTCCTTAATAAC
wssv-m2->gi426202315:225865-225921
AGAAGAGGACTTTGGGGTAGACATTTTCTTCCTCTCCCCTTCCAGGTCCTTAATAAC
wssv-m2->gi19481591:194131-194187
AGAAGAGGACTTTGGGGTAGACATTTTCTTCCTCTCCCCTTCCAGGTCCTTAATAAC

****

MUSCLE (3.8) multiple sequence alignment

wssv-m3->gi58866698:285122-285215
AAAATTTCTTGACGATAAGAGGAGGCAGTAGGTGAGGCTGCTTGTTTGATGTGTCAGCCA
wssv-m3->gi426202315:236397-236490
AAAATTTCTTGACGATAAGAGGAGGCAGTAGGTGAGGCTGCTTGTTTGATGTGTCAGCCA
wssv-m3->gi721172032:241024-241117
AAAATTTCTTGACGATAAGAGGAGGCAGTAGGTGAGGCTGCTTGTTTGATGTGTCAGCCA

wssv-m3->gi19481591:271018-271111
AAAATTTCTTGACGATAAGAGGAGGCAGTAGGTGAGGCTGCTTGTTTGATGTGTCAGCCA

****

wssv-m3->gi58866698:285122-285215
CATCTGCGTCATACATTATATTTCCAAGAATTTT
wssv-m3->gi426202315:236397-236490
CATCTGCGTCATACATTATATTTCCAAGAATTTT
wssv-m3->gi721172032:241024-241117
CATCTGCGTCATACATTATATTTCCAAGAATTTT
wssv-m3->gi19481591:271018-271111
CATCTGCGTCATACATTATATTTCCAAGAATTTT

****

MUSCLE (3.8) multiple sequence alignment

wssv-miR-211-a->gi426202315:272850-27294
GCCCTGTTCCAGAAATGGCTGTCCAGAAATCTGGGTCGGACAGATTCCAGAAACGTTTCT
wssv-miR-211-a->gi58866698:28422-28515
GCCCTGTTCCAGAAATGGCTGTCCAGAAATCTGGGTCGGACAGATTCCAGAAACGTTTCT
wssv-miR-211-a->gi721172032:277471-27756
GCCCTGTTCCAGAAATGGCTGTCCAGAAATCTGGGTCGGACAGATTCCAGAAACGTTTCT
wssv-miR-211-a->gi19481591:173-266
GCCCTGTTCCAGAAATGGCTGTCCAGAAATCTGGGTCGGACAGATTCCAGAAACGTTTCT

****

wssv-miR-211-a->gi426202315:272850-27294
AACCATTTCTGGAACAGTCATTTCTGGAAAGGGT
wssv-miR-211-a->gi58866698:28422-28515
AACCATTTCTGGAACAGTCATTTCTGGAAAGGGT
wssv-miR-211-a->gi721172032:277471-27756
AACCATTTCTGGAACAGTCATTTCTGGAAAGGGT
wssv-miR-211-a->gi19481591:173-266
AACCATTTCTGGAACAGTCATTTCTGGAAAGGGT

****

MUSCLE (3.8) multiple sequence alignment

wssv-miR-211-b->gi426202315:273818-27390
CTGTTCCAGAAATGGCTGTCCAGAAATCTGGGTCGGCCAGATTCCAGAAACGTTTCTAAC
wssv-miR-211-b->gi58866698:29716-29803
CTGTTCCAGAAATGGCTGTCCAGAAATCTGGGTCGGCCAGATTCCAGAAACGTTTCTAAC
wssv-miR-211-b->gi19481591:1141-1228
CTGTTCCAGAAATGGCTGTCCAGAAATCTGGGTCGGCCAGATTCCAGAAACGTTTCTAAC
wssv-miR-211-b->gi721172032:278439-27852
CTGTTCCAGAAATGGCTGTCCAGAAATCTGGGTCGGCCAGATTCCAGAAACGTTTCTAAC

****

wssv-miR-211-b->gi426202315:273818-27390
CATTTCTGGAACAGTCATTTCTGGAAAG
wssv-miR-211-b->gi58866698:29716-29803
CATTTCTGGAACAGTCATTTCTGGAAAG
wssv-miR-211-b->gi19481591:1141-1228
CATTTCTGGAACAGTCATTTCTGGAAAG
wssv-miR-211-b->gi721172032:278439-27852
CATTTCTGGAACAGTCATTTCTGGAAAG

****

MUSCLE (3.8) multiple sequence alignment

wssv-miR-211-c->gi19481591:820-903
TCCAGAAATGGCTGTCCAGAAATCTGGGTCGGCCAGATTCCAGAAACGATTCTAACCATT
wssv-miR-211-c->gi426202315:273497-27358
TCCAGAAATGGCTGTCCAGAAATCTGGGTCGGCCAGATTCCAGAAACGATTCTAACCATT
wssv-miR-211-c->gi721172032:278118-27820
TCCAGAAATGGCTGTCCAGAAATCTGGGTCGGCCAGATTCCAGAAACGATTCTAACCATT
wssv-miR-211-c->gi58866698:29395-29478
TCCAGAAATGGCTGTCCAGAAATCTGGGTCGGCCAGATTCCAGAAACGATTCTAACCATT

****

wssv-miR-211-c->gi19481591:820-903
TCTGGAACAGTCATTTCTGGAAAG
wssv-miR-211-c->gi426202315:273497-27358
TCTGGAACAGTCATTTCTGGAAAG
wssv-miR-211-c->gi721172032:278118-27820
TCTGGAACAGTCATTTCTGGAAAG
wssv-miR-211-c->gi58866698:29395-29478
TCTGGAACAGTCATTTCTGGAAAG

```
************************

MUSCLE (3.8) multiple sequence alignment


wssv-miR-N1->gi19481591:231993-232086
GCTTGAGTATTGGCAGGTCTCTTCTAGGTAGAAGGAGAGAGAGTGCTTGTTGTGTTGCTC
wssv-miR-N1->gi721172032:258800-258893
GCTTGAGTATTGGCAGGTCTCTTCTAGGTAGAAGGAGAGAGAGTGCTTGTTGTGTTGCTC
wssv-miR-N1->gi426202315:263397-263490
GCTTGAGTATTGGCAGGTCTCTTCTAGGTAGAAGGAGAGAGAGTGCTTGTTGTGTTGCTC
wssv-miR-N1->gi58866698:202307-202400
GCTTGAGTATTGGCAGGTCTCTTCTAGGTAGAAGGAGAGAGAGTGCTTGTTGTGTTGCTC

************************************************************

wssv-miR-N1->gi19481591:231993-232086
TTCTGCTGGCTTGCAGAGAACTGATGCTTCTGGC
wssv-miR-N1->gi721172032:258800-258893
TTCTGCTGGCTTGCAGAGAACTGATGCTTCTGGC
wssv-miR-N1->gi426202315:263397-263490
TTCTGCTGGCTTGCAGAGAACTGATGCTTCTGGC
wssv-miR-N1->gi58866698:202307-202400
TTCTGCTGGCTTGCAGAGAACTGATGCTTCTGGC

**********************************

MUSCLE (3.8) multiple sequence alignment


wssv-m6->gi426202315:173896-173966
TTCCCCGGTATGTTTTTCAAGCTGCGAGTGCCCGCAAAGCCTCTCTTGCCTGGAGAGACT
wssv-m6->gi19481591:207488-207558
TTCCCCGGTATGTTTTTCAAGCTGCGAGTGCCCGCAAAGCCTCTCTTGCCTGGAGAGACT
wssv-m6->gi721172032:178523-178593
TTCCCCGGTATGTTTTTCAAGCTGCGAGTGCCCGCAAAGCCTCTCTTGCCTGGAGAGACT
wssv-m6->gi58866698:222694-222764
TTCCCCGGTATGTTTTTCAAGCTGCGAGTGCCCGCAAAGCCTCTCTTGCCTGGAGAGACT

************************************************************

wssv-m6->gi426202315:173896-173966     ATAGGGGAATA
wssv-m6->gi19481591:207488-207558      ATAGGGGAATA
wssv-m6->gi721172032:178523-178593     ATAGGGGAATA
```

```
wssv-m6->gi58866698:222694-222764      ATAGGGGAATA
                                       ***********

MUSCLE (3.8) multiple sequence alignment


wssv-m7->gi58866698:259013-259097
TTTTAATAACCATTTCATTGCATCCGGAAAGGGTAATGAAATATCATTCATCGATCCTCC
wssv-m7->gi426202315:15003-15087
TTTTAATAACCATTTCATTGCATCCGGAAAGGGTAATGAAATATCATTCATCGATCCTCC
wssv-m7->gi721172032:10394-10478
TTTTAATAACCATTTCATTGCATCCGGAAAGGGTAATGAAATATCATTCATCGATCCTCC
wssv-m7->gi19481591:288700-288784
TTTTAATAACCATTTCATTGCATCCGGAAAGGGTAATGAAATATCATTCATCGATCCTCC

************************************************************

wssv-m7->gi58866698:259013-259097     GAATGAAAGGTTGTTGATGGAGATG
wssv-m7->gi426202315:15003-15087      GAATGAAAGGTTGTTGATGGAGATG
wssv-m7->gi721172032:10394-10478      GAATGAAAGGTTGTTGATGGAGATG
wssv-m7->gi19481591:288700-288784     GAATGAAAGGTTGTTGATGGAGATG
                                      *************************

MUSCLE (3.8) multiple sequence alignment


wssv-m8->gi426202315:54512-54606
TCTGGGGATTTATTAGCGTCTCTTGAGTCTTGTAGTAATTTCGTTTGCCGGGATGTGCGG
wssv-m8->gi721172032:49896-49990
TCTGGGGATTTATTAGCGTCTCTTGAGTCTTGTAGTAATTTCGTTTGCCGGGATGTGCGG
wssv-m8->gi19481591:22069-22163
TCTGGGGATTTATTAGCGTCTCTTGAGTCTTGTAGTAATTTCGTTTGCCGGGATGTGCGG
wssv-m8->gi58866698:286613-286707
TCTGGGGATTTATTAGCGTCTCTTGAGTCTTGTAGTAATTTCGTTTGCCGGGATGTGCGG

************************************************************

wssv-m8->gi426202315:54512-54606
TACTATTTTTATAGAGGACGTTTTTAAATTTCAAA
wssv-m8->gi721172032:49896-49990
TACTATTTTTATAGAGGACGTTTTTAAATTTCAAA
wssv-m8->gi19481591:22069-22163
TACTATTTTTATAGAGGACGTTTTTAAATTTCAAA
wssv-m8->gi58866698:286613-286707
```

TACTATTTTTATAGAGGACGTTTTTAAATTTCAAA

****

MUSCLE (3.8) multiple sequence alignment

wssv-m9->gi721172032:251521-251611
GGCGGTGCTTTTCCTCCCACCTCTCCATTATTCTTCTTTAGTCTCTTCATGGGCCTAAGA
wssv-m9->gi426202315:246894-246984
GGCGGTGCTTTTCCTCCCACCTCTCCATTATTCTTCTTTAGTCTCTTCATGGGCCTAAGA
wssv-m9->gi58866698:2652-2742
GGCGGTGCTTTTCCTCCCACCTCTCCATTATTCTTCTTTAGTCTCTTCATGGGCCTAAGA
wssv-m9->gi19481591:281516-281606
GGCGGTGCTTTTCCTCCCACCTCTCCATTATTCTTCTTTAGTCTCTTCATGGGCCTAAGA

****

wssv-m9->gi721172032:251521-251611
GAATAAGGAGAATCTGAGGGAGGGGATCGGC
wssv-m9->gi426202315:246894-246984
GAATAAGGAGAATCTGAGGGAGGGGATCGGC
wssv-m9->gi58866698:2652-2742
GAATAAGGAGAATCTGAGGGAGGGGATCGGC
wssv-m9->gi19481591:281516-281606
GAATAAGGAGAATCTGAGGGAGGGGATCGGC
****
MUSCLE (3.8) multiple sequence alignment

wssv-m10->gi721172032:252819-252885
GTGACTGGAGTGTTGATTGGGTATAGAGATTCTTGGTGCTTCTTTTCCCTTTCTGATGCT
wssv-m10->gi19481591:282814-282880
GTGACTGGAGTGTTGATTGGGTATAGAGATTCTTGGTGCTTCTTTTCCCTTTCTGATGCT
wssv-m10->gi58866698:3950-4016
GTGACTGGAGTGTTGATTGGGTATAGAGATTCTTGGTGCTTCTTTTCCCTTTCTGATGCT
wssv-m10->gi426202315:248192-248258
GTGACTGGAGTGTTGATTGGGTATAGAGATTCTTGGTGCTTCTTTTCCCTTTCTGATGCT

****

wssv-m10->gi721172032:252819-252885   TCATCAC
wssv-m10->gi19481591:282814-282880   TCATCAC
wssv-m10->gi58866698:3950-4016   TCATCAC

wssv-m10->gi426202315:248192-248258 TCATCAC
****

MUSCLE (3.8) multiple sequence alignment

wssv-m11->gi19481591:230394-230488
GCTGGGAGTCTAGGTTTCTTCCCATTCTTGTTACCCCAGCTCTTGTCCAGATCAGGGTTA
wssv-m11->gi58866698:200707-200801
GCTGGGAGTCTAGGTTTCTTCCCATTCTTGTTACCCCAGCTCTTGTCCAGATCAGGGTTA
wssv-m11->gi721172032:257200-257294
GCTGGGAGTCTAGGTTTCTTCCCATTCTTGTTACCCCAGCTCTTGTCCAGATCAGGGTTA
wssv-m11->gi426202315:261797-261891
GCTGGGAGTCTAGGTTTCTTCCCATTCTTGTTACCCCAGCTCTTGTCCAGATCAGGGTTA

****

wssv-m11->gi19481591:230394-230488
ACATTATCGTGGAACAGAAACTTTGACTTGTCATT
wssv-m11->gi58866698:200707-200801
ACATTATCGTGGAACAGAAACTTTGACTTGTCATT
wssv-m11->gi721172032:257200-257294
ACATTATCGTGGAACAGAAACTTTGACTTGTCATT
wssv-m11->gi426202315:261797-261891
ACATTATCGTGGAACAGAAACTTTGACTTGTCATT

****

MUSCLE (3.8) multiple sequence alignment

wssv-m12->gi19481591:118951-119050
TGGGGGTTTCGTTTGTGGTTGCTCTCCTCCTTCATCATCTTCACTATCCGAAGCTGAAGA
wssv-m12->gi58866698:89419-89518
TGGGGGTTTCGTTTGTGGTTGCTCTCCTCCTTCATCATCTTCACTATCCGAAGCTGAAGA
wssv-m12->gi426202315:150367-150466
TGGGGGTTTCGTTTGTGGTTGCTCTCCTCCTTCATCATCTTCACTATCCGAAGCTGAAGA
wssv-m12->gi721172032:145751-145850
TGGGGGTTTCGTTTGTGGTTGCTCTCCTCCTTCATCATCTTCACTATCCGAAGCTGAAGA

****

wssv-m12->gi19481591:118951-119050
AGAAGATGAGGAAGAAGGAAGGGGTCTCTTGACACCTCTA

wssv-m12->gi58866698:89419-89518
AGAAGATGAGGAAGAAGGAAGGGGTCTCTTGACACCTCTA
wssv-m12->gi426202315:150367-150466
AGAAGATGAGGAAGAAGGAAGGGGTCTCTTGACACCTCTA
wssv-m12->gi721172032:145751-145850
AGAAGATGAGGAAGAAGGAAGGGGTCTCTTGACACCTCTA

****

Text S3

WSSV primary miRNA (pri-miRNA) sequences.

>Unigene31474_All pri-miR(wssv-m2) size 5334
CCCCAGCCACAGCAGCAGCAGTAACAGCAAGCAGGCATCAGTCAGCCAGGTTCAGCATAACAAATCAAATT
GGCAGCAAATAGCAGCAGCAGCAGCAGCAGCAACCACCACAGCCGCCCAATAATATTCCTCCTCCCCCTACT
CCTCAACAACAATCACCCAGTAATATTCCTCCTCCCCAGCAGCAGCAGCAGCAGCCCTTTCCGGTTCAACTCA
TTTCTAGTCCCCCTCCTCCTCCTCCTATACCTAATACTGCTCCTTCTCCACCTATTTCCCGTGTAAGATTTGACTC
TCGTTCTACTACCCCTCAACCTCCACCTACACCAGTTCTACCCAAGCCTACTCCTCTTCCTCCTCCGTCTACAGC
AAGAGCAGAAGAAGAAAACGCTACTGATATGTCCTTTACTGATATAGACTCTGAGCTTGGCAGTATTGATTTT
GATCTTCCTCCCGCTACTCCAGGGAGGAACGTTGAAGAGATAATAAAAGCGCAACGTCAAGCTGTCAAGGA
AACGGGAGTCAGAGGAGAAGAAGAAGAAGAAGAGGAGGCATTTATTGCACCAATTATTCGTCAACCGCGT
ACACCAGGAAATTTTAGAGATGAACTTTTAGATGTCAATGAATCCATCTATGGCTCAGACATTGAACCAGCAG
CAGCAGCAGCAGCGTTTGACTGGGATATGGGGTTAGACGATTTAAATGGGGATGAACCATATGAATTTGAAT
AAAACACAACATGTAAAACATATAATTTTTATTGGTAAAAATAAAGGTATACATTTAATGCTTCTTTGTTTTCTT
TGCCAAAGATTTCTTGTACTTTCCTCTAATGTCTTTCATGGCATCAATTTTCTTAATCATTACATGCATAAAGTCT
TGGATTGCAGCAGCATCAGTATCAGTTGAAGAAGAGGTAGAAGTAGTAGAACCATCATCAGCAGCAGCAGC
AGCATTGTCGTCAGCGGTTTCATCTTTGGACGAGCAGGATTTGATACTCTTAAATGTATAGTTTACAAAAACT
GCACCATAGTCATCCAAAGCATTTCCGTCCTGGTACTCTTCATGGGTCAATTTACAAGTATACAATGGGTCATT
TAAAGATGAAGTATAAATACATAGGTTCAAATCACTAGGTGAAGTTGCAGTCATATTCTTATTGAGCAAATATG
TAGACTCGCCGGTAAAGGCGCTAACACTCTTGCATACCTTCTTTAGACCGTTAAATCTAATCTTCCTATTCCTAT
CAATAATATTCCTACACAACTTTGTTTTAGGTGCAGTTTCAGCTACAAGTTTTACAGCGTGTCTATGGTTAACA
ATAAAACTGCAAAGTTTGGGTGGTACAACAAATCCACACCTCCACATGTTCCTAGATGTCAAAAACATGTTCT
CGTTCCAGATGCTCTTTTTCTTTGTAGAAGAAAGGGAGCAAGATAGAGCCAGAACAGAAGATGAAGGAGC
TACAATAGACGAGACGGAAGAGACTGAATTTGAACTAGAAGGAGTTTGTACACACATGCCATTATTATTGCT
CATACCAGATGAGATCTTCTTCCTATTGAATTTTGTCTTTTTCTTTTCAGTGAGAGAAGAGCTAGGAGAAATAT
TATAGAATGGAAGAACCTTATTCTTATTAATATTGAAAAAGGTGTCATGCTTAGACAAATGCAAAATATCCTTC
TCTAGCTTGTCTGTTGTAGTAGAATCAACATCCTCATCGTTTAATGAATCATATTTGGGTGAAATTGTCAAAAT
GCCCTTTCCGTCTATGGTGTTAACGTACAATTTGCCACATGAGGCCTTATCTAGTCCTGCGCTTGAAAAATCA
ACAGGTATACTAAAGGGGCCTACAATATGTGAATCCACACGCGTACTGACTCCAAAAACAGTTTCATATTTAT
CATCTGTTCCATTGATTTTCCTATTGATTTTGACATCGCTAAACACAAACCTTGAGACCTCATCAAGGGTATTG
ATTGTGCTAGAATCCTTCAAGAAACCGGGAGGGATTTTCTTGTTCATCCAGCTTATTCCAGAGATCAAACTAT
TAATTGCAATAGAAACTACCATCGAATTTTCACACAAACTTTTCACAAATTTTCTAATGACTTGATCCCCAATAC

TGTTGAATTTTTGACCCTTCTTTCTCCCTCCCTTGCGAACTTGCTTCTGTTGTTCTACGTCCATACTTTCTTCCA
TTTTATCCTCCTCTCCTTCTCCTTCCTCCTCTTCCTCCACAATATCGGTTTCTTCGTTATCATTATTGTTGTTGATG
TACTGGAGCACATCGCAAATCCTGTTCCTGGTCATTACAGTCAGGTACCACAGGCAAGTGGAAGAAAAGTG
AACGTCATGGGTATAGTTGTCTCCTTCCAATTCTCTCTTAATCAGATTGAACAAGAACATTGGTACAAACTTGC
TAAACGCATGGTATTCTTCATATGTCCTACGAATAGCATTAACGCTCACCAGTACCTTTGCATACTTGTTTTTGC
GCTTAGGGGTGTTAACGTTTTCTAGGCTCCAACTCTTAATATTCTCCAGTACTTCTTCAAAGTAAGCGATCGG
GTCCTCCAAGAACTCGGCAAACAATTCCCCTTGGTGAGACAAAACATCCATTTCATTTTCCTCAATATCTACA
GCTTGAGACCTGCATTTCTCATACAAGCGAGTTACCTCCCTCTCATATCTAGTCAGTCTAGTGAATGAGCTCCT
CATGATTTCATTTTCAAGATCTTCAAAAGTCTTGCACATTCTCTTAAATGTGCTGGAAGCATCAGACAGTCCAC
AAAGACGTTGACCGAATATTTCAGACAAATATGAAGCCGAGCCATTGACATTACTGCGTGGCATTTTTATGAA
ACTGAGGGGAATTAAATTGATAGTTTCCTTGTCATGACCAGCTTGTTGTTGTTGTTCTGGCGTAGACGATGAA
GAAGGAGATGATGATTCTTGTGCAACCTTTTCCCCTTCATCATTTTTCATCATTTTTGTTGATGGTTCTTGGTG
TTCATCGTTAGATGATGAAGCACTAGAGGTACGCTTGCGCTTCATTTTTGAGGGCTTTTCTGATGCCATTTTG
AAGGAGAATACGTTAGGTATGACTGTGCCAGAATGGAGTTTCGATTTCACCCACGCAAATAGTGCATTATTCA
CATTAGATTCCGTGTTGGTTTTGAAGCACGAGGAAGAAATGTGCTTGTTTCCATACAGACTCTTCTTTTGTCC
TTCATATTTACCCTTAATGTTGGTAGAGTTGGCGTTAAACATTCCTCCAAATATTTCAGACTTGCAAATATCTTG
AGCCATCTGATGTGCCACAATTGATGTGACAACCTCATCAAACAACAAACCCTTAGCAAATTGTTGACTTTCT
TCAGTCCAAATGGTTGGACGGCGACGGATACTATCGTCCATGGTATTCAAACACTCTTGGAGTTTGTTGGTG
AATACACGGTAACTGCTCATAGATGATGGATTATTACAAATTTTAGCAAGAGAATTTTGCATAGTTTCATACAA
AATATTTTCAGTTCTCTCGTCGTTTGGGGCCATGAAGGAATTTTTCACTGTAGCTTCAGTTTGGGGGCATAAT
GCAGTAGCGTCAACGGCATCGTCATCATCACCATCATCTTCGTTATTGATACTACTGCTACTATTGTTACCCCCA
GAAGTAATTTTGAGTGCAGGTTCATTCTTAACCCTTCTCATTTGTTTCATAGTAGAAAGCTTGGCTAATTTCTT
CATAACAAGAGCATCTTCTGAACCTTCATACTTGCTACTTTCGTCTTCATCAATTTCTTCCTCTTCTTCCTCCCC
CTCCACAACTGCGTTACCTTCGTATGGTGTAGGAGGAGGAACTGTTGGAGAGGAAGACGATGACGATGAAT
TTTCTCCATCTTCTTCCGTCTTGATGGGGGAAGGACTAGCATCAAAAAGACGCCTGTCAGATTCGACCACAA
TATTCATAGAACGTTCTTCATGTTCTTCATTATTAACAGTAACATCGATGCAGTCAGCAAAACAGCACAAGTCA
TTCATATCACTAATTGCACATTCGTTAGAAAATCCACTGTCAGATGTGACCACACCGTTTGCGTTGCACCAACT
AGCCGTGTTTTCATACTCGGTTATCATATAGAGCAATTGTTCTTTCCCGTCTCCACTGTAGTCGTACACGGCCA
CATGAGGTGTAATCGCTGGTGCGTCATTCATGCGCTTCTTCCTCTCCAAATCTTCGGTGTGACACCTCATTCTG
GCATCATTTTCCTCTTCGTCAAAAGACTGCGTCTCAATTGAAGTGATGTAAAAATCTTCCAAGTTGTCATATTC
TGTACGTCCAAACATGCACAGAACAGGAAGCTTGGCCATCCTCAAACCAGTCATGTTTAGGGTTTGAATACC
TGCATTCATGTCGAAAACTGGTACATATTTAGGTAGAGATCTTTCTAGCCCAACACCAGTCAAGAAAACTGCA
TATGTATTTCCATCTTCTGTAGAAGTTTCGAACCCAACAAGAGGATAGGTTGTTTCAAGCATAAAACAATCCC
TGGGTTCTTCAGAGAAAACTTGGTTGTTGTTGTTCTCAAATGAGGAGACGTTGTGTTTTTTGATACTCATTGA
CACTGCATACTTGCCTCCAATTCTTGAACAGTTGGCGTTTAACTTGAGACCTTTGCACACTCGCCCCTTGGAG
TAGATTTCTAAATCCTCGACAGAAAACACTTTTTCTGGGCTGGAAGTTACACTGTTCTTGGCTTCACTTTCAA
TCTTGGTTCTGCGCACACTTTCAAGTACATTTTCAATTTCCTTGATGGTCGATTCAATGGAATTATCTGCAGTG
CTCTTAACGGCCTGATACTCGTTGATAATATCGTCCAACATAGCCTTGTCCAGTTTTTGTCCATCAGAAGAGGA
CTTTGGGGTAGACATTTTCTTCCTCTCCCCTTCCAGGTCCTTAATAACCTTTGATGCAAGAGTAGCCTTGGCAT
GAAGATTGGCAAAGGAATGCTCTGCAACTTTCTTCACTGATGAATACAACTGGGACATTTTTGAGGGGGTA
GAACTGGTAGTTCCTTCAAAATCCATTTTAGGGACGATTTCAGTAGGTTTTCTTGAATAATATCACAAGAGGA
GAGGATGTTCTCAGTACTTGAAATGTCTTGAGAATGATGTCTGTGGTGTGCTGGTGAGCTCTATTTATACCCG
ATTTTGAGGGGGGAGCGGGAGGGGACATTTTTTATTAATGATGGTGACAAGGTGCGAGAATGCGTGTGTG
TGTTTACACTTCTGGACACCTTTCAGGCAAGAAATTTTACTCTTCCTCCATCTCAA

>Unigene33547_All pri-miR(wssv-m3) size 1705
GGTCCACCCTCTAAACTCGAGTGACGCAGAAAAATTTTTGAAAAATTTTTGATGAAGAGATTGAGTAAAATT
TCTTGACGATAAGAGGAGGCAGTAGGTGAGGCTGCTTGTTTGATGTGTCAGCCACATCTGCGTCATACATTA
TATTTCCAAGAATTTTGCTGACGTCAATGGACCATAAAAGGCTTTGTACGTCCAGAGACAAGTTTTAGTCTGA
TAGATTTCTTAAAAAAAAAGAGGGTGGGAGGTTTGTTTTTGTGGGTTCTGTGTGTGTATAAAAGATAGGTGC
AAAGGTAGAGAATCATCATATGGACAAAATCTGTCCATGAGACCTCAGTAGAGAACGCACCATGGTTGCTTC
AACTCCGTGTCCAGGCCCAGGACCAGTTCCAACCCAAGAACTTCTTTCTACAAACTTTCTTGAAGCTCACAA
GCTTGTCGTGGAACTTCTTCTCCCGTCCTACAGTAGTGATGTAGTTTATTGTGACTCTGAGACGTACACCAAA
CCTATACCGATTTTTGGGAACAAGAGTATAGTTTCTACCATTGGAGACTATGTCTTATCAAACCCCAATGAAG
ATGTGAGTTACCAAATGGTTTCTTCCGTCTTAGAAAAATTTCCCTTGCTATTCCACTGCACTTATAAGACGAAT
GAAGAAGATAAAGGTATTCCTCTGTGGAAGAAGTTGTACAACAAAAGAAAATTCAAACTCCTCAACTCATTG
TTGGTTCATAACAACAAGAACTGGACTCCTGTTCCAGCTATCCCGTTTGACAGGGAGAATATATGTGATGCTT
CAGGAAGGAGTGTTCTTATGAGTGAAATAATGTCCACGTCAACTTTTCAGACAATTTGCAAAAACAACACAC
ATTACTTGTTTGATATGTTAAATATGGAACGTGGCAAACAAGGAGGGAGTTTTCTTCACTTCTTTGCATCTAG
GAAGAATTCTTTTACTAACTTTGAAAATGAAGAAATGGACTCTCATGTGCTCAGTAACATAGCGAAATTCATA
TGCAATGAAAAGGAAAAACTAGACTCTTTCATACCTGCCAACGGAAAAATACCATGCCCTGATAAAACTAAT
GATGAAGGGTACATCCCGCTGGAAATAGCAATTATGGAAGACAATTACCCTGCATTGCTATATCTCGTTTGTA
GGTATGGAGCATCTTGGGCAAACACATACGGGGATCATAATGAATCTCTCAAAGCGTTTGCAATAAGAAATG
ATGCAAAAGATTGTCTGGAAATTATAGAGTTTATAAGTGATCACTACAGTTTCAACAAAAATGTGACGAAGG
AAGAATTTGTTAAAGAGAAGACTGTAGAATGTGTTGGATGTTTATATGATATTGAAGACGAGAAACGTTGTT
ACAAACTCCCATGTGGACATTTCATGCATACATTTTGCTTGTCTAATAAGTGTTCTAAAGCTAACTTTAGATGT
GTTAAATGTTTCCAAACCTTTGATGACACAATTTTTAGAAAATGTCCCCCAACTATACAATGGAAAATGGGTAT
AAACCAAACGACTAACCATAAGGAAATGGATTTGTTCAATCGTGCATTTGACACATATTTAGATTTTATTTGCT
CATATAACGTCAAATTAGACAAAAAATCAAAACCTAAACACAAACCTGAAAACAAAAAGGTGGAAGAAGAA
CTAGCAAAAAGGACAGCAGAAATTGAAGAGGCCAT
>Unigene39470_All pri-miR(wssv-m5)size 2926
ATATAAAACATTAGTTTATTGGTATCTTATTTACACAATATACAGAAAATGTCACATATCAACCAATCAATCAGTC
ATCCATGTCGTTGCGAACCCTTTCCCTGCACTTGGGAACATCACATGAAGCGTCTCTGCAAACAATGTAGTGA
TATGCGAGCATGGATGTACATGACCTCCTTACGATACATCTATTACAATATTGTCCATGTCTAATTACAGTCTTC
GCCCATTTGCACATGGCAGTACATACATCCATATTACAAAGAAAGCGTGGGAAATGTTACTACAGAATTTGG
GGACTGTTCCTCTACGCTTTGCAAGAGAGAAGGATCCCTTGAACCACTCCTGGACAACATTACAAGTCTGTC
CCTTGTTATATGATGGAACAAGAGGGGATAGATATTTGGCCATATACTCATAAAATGGATGAAAGACTTTATCC
TCTTCGTAATTGTAAGAATTGGGAATACTTGGATCATCTTCGTCACATTCTAAGGGGAACGTGGGAGGGACA
AATCCATTAGGCACTACTACATTCGCACCTCCTGTGAAGCAGGTAGGAATTGGGGTGTTAGACAGGGGAGA
TGTTCCTGTATTTGCACTCATTCTAGAATTTCCACCTCCTCTGGAAGGCGAATAAAGCCACATCATGTCCAATT
CTGGTACATCATTCGTTAAAGGAGTAATTACCAATCTTGGGGGAGGGTTGGGAAGTGGGTTGGTATTCACAC
CCACTGATGAAGTCTCTATGGGGTTGGTATTTGTTCCCATTGATTGGAATTCTTGCATATCAGTATTTGTTCCC
ATCGTTTGGAATTCTTGCACATCAGTGCTTGTTCCCATATTACTAGTGTCAGGCGTGAGACGCCTCTTCTTACT
GGCACTCCCATTGGTCTTTTGTTCTTCCCTCTTGTGGAACACTACAGTATCAAAATTTTCTCCTGTTGCATACT
CTGACGCATCGCCATGTATTCCTCCATCATCTTTATCAAAGCATACGACAACTCCATTAACTATCCAACAAAGG
TTAGTATAGAATGTCATATCACTGCACAGTGTGTCTGCACGAGCAATCAGATTGGGGAAAGCTTTGTTCAATA
CTCTTGAAACAAACATCCTTGGGACCTTGTATTCATCTCCATGTTCAGCCAAGAATCTTTGTTCCAAGTGAGC
CAAAAATACACCTCCCCTTTTACCGGGAGGGGCCATGCATACATTCTCGTGAATCCATTTCCTCAAAGCCTTT
CTCCCCTTAAGGAATTGACATTTCTTGAACCCTTTCTTATTATGCATGGCAGGTGTGGGAGGAGGAAATACAT

AGGAAGAAGTATACACATCTATGGATGGGGTGGGCAATTCAGCGCAAAATTGAGGAGGGGCGACACCGCA
CATATCGATACTAGGGTCAACTTTATAAAGGGAAATGTTTGCCCTTGTTTTATAATCTGCTGCGACAGGAGGT
TCTCCTCCTACATATAGGTGAGGGAGAGGATCTTTCATCATCTTTTTGAGTTTCTTAATTTCCTTCCTCTTTTTC
TTTTCCATTTTTTTCAATTCAGCTTCAGAGATCCCGACAGGGATAGAAGAAGAGGCGGCAATAGACGGTGCT
GATGCTGCTGCTGCTCCTTCTTCGTCACATTCATCCGAATCAGTATCCATGATGGATCGAGGAGTAGGAAGTG
GTGTTGTTGTAGAGGGAGGAGGGAAAGGGGCAGTAGAAGTTGATGGCATGTTATCTGAATCACCAAACATA
TCAAAGTTTGATCCAAAACATTCTTCTTCTCCCTTGTTATCGTCCTCATCGTTATCACTAAAAGATACAGTTTCC
ATGTACGAAAAAATGGGGGGACGGTTCGAGACTTCTTGCTTGACATCTTTTTCGTCTTCTTCGTCATCATCAT
CGTCATCATCCCACAAAGACGATGGAGAGGTCGTCGAGGACATAGATGGAGGGGGTGTGGGGTAGTAATT
GTATTGGTGGCATTGTTGTTGAAATGAATCAGAAAATTGATAGTCAGAAGTGAATGGGGAGGAAGTATAGA
GAGGAGAAGGAGCATAAGGGCGATAGGGGGATTGGTGAGGGAGAGCAGTAGAGAATTTGTGTTGTTTTT
TGTATTGCTGTGGTTTTAGAAAGACTCCCGAATCAGAAATTTGAGCGGAAGTTGATGAGTCGACTTCTTGTC
CAGAGGGGAGGCTTGAAGGGTTGATGACGTCATCAGAGACTGGAGAGAAGTTTGCAGACACTCCCATTTC
TTCGTCCTCACTCTTCATAAGGGCGAACATCTGCCTCTCAGTCACGGGGCGTTGGGGCATACTTGCACACAA
GGCAGAGTAGTTAAAATCGTCTCCTTGGAACTCCATGGCTTTAACTTCTCTCATCACAATGTTGGTGGTGAAG
CGGGTCTTCTGGCAAAAGCTCACCAGTGCCTTCACAACCTTCGCACCAACACAAGCAGCCGGGGACGAAG
TTTGGGCGCCTTCGGGAGATAGAGGTCCGGACATCACCTTATCCAAGAGATCGGCTGCAGCGTTCAGGTGA
GGCGGGGCCATTGTGAAACATCCATAGTCTCCGTACTGGTTGGAGATTTCTGTCAAAGAGTAAAGGAATTG
GACTTAGAATATTTTTCACTTCAACCAACAAAATATTCAGCATAACATAGAAAATTTCAAAGTCCAAAGTGTAT
AGTGAGAACTTGTGCTTACCTTGTATGAATTGGAAGATCTTCTCGTGCTCCTCGAATAGATGGAGCAAGTGTT
TCTTCATATCAGACCGGCGAGAGCTAGTCGAGACGTATGAAGTTAGTTCCTTTAGTTCCTCAGAACTAAGTAA
CTTAGAGTCCAACTTGGTGTAAGGACTTGGTGCGGCGGGGGCTGGAGAGGCCATTCTTGTAGCTTGAGTAT
TGGCAGGTCTCTTCTAGGTAGAAG
>Unigene33544_All pri-miR(wssv-m6) size 7910
TGAATAAAAATACAAGTAATTTTATACCATCTTTTATTTTTCTAATCCTTTGAAATGTATCTTGTTACCTGACTCA
TTACAAATTTCTCATCATCCCTAAAGAATGTGTACAAATCATCATTAGCAAATGTACAAATAAAGTTAGTCAAA
AACACACAAATATTAATCTTCATGTTGTAAGGAATGTTGGACACAACAGTACCCAGAACACTGTTCATGATCA
TGCTGTTACTCTGCAACAATTCCTTACTTTCAATAGCTCCGGCCACCCTCTTAATTACACCAACCAGAATCCTG
TTAAGGCGATCAACGTAATCGGAAAAGGAGAGGTCAAATTCTTTGGGGGATTTCTTACTCTTAAGAATGACG
CTCAGTTTTTCGTCGCTATCGTCCTTATATTTCTCCACAACATTCTGCATCTCCTTAAAATGATAGAGGTGGTCA
ACCAACATCTTGCGCACAGGCTGATTAATTGCATCGATAACAATTTTAGTAGTGATGCTCTTTATGCGTTCAAT
GGGAAGAAGGCTCTTGAACTTGCTGTCTTGAGCGATCCCAAACATTGAAAAAATTTCAGCAATAGCTTCAG
GGGACTTTTCTTCAACTTCTTGCTTGACCTTCTCATCAGGAACAGTGGGGTCGCTCCGCTCCAGATCCTTCAC
GAATTCTAGAAGTTGTTCTTCCTTGGTCTTGGTGTGTTTATTGGATTTGCTCTTCTTCTTAGAAGATGTGGATG
AACTCTTGTCTTCAAAACTGGGTGGTTCCGATTTAGATTTCTTTGTGAAAGGTTGTTGCCTTTCTTCCTCCACT
TCCTCCTCAAAACACCTCTTGTTAGTTCCCATCTTTGATGTCTGAGCCATTTTTATTATGACACAAACCTATTCC
ACAAAAGATAGTTTTCCTTAATTTCACGGAGAAATTTAGTTGAACACGATCTCTGCGCACTTAGAGTACGTAC
AAGATCTAAAATGAGAACATACACAGGTCCGGTGAGATTTGCTACTGCATCTAGACAATCTATCCTCGTATCG
TTTACACCAAATTTTTTCACTGCATTGGCTCTTTCGCTTAAAATTTCCTCAAAATGAGCCCAGGACTTTTCGAA
TGAAGAACCGTATTTTCTAAGTAGAGGGGTTAAACATGTACCATATAGACCATGGTATTGTGTTGAATGGGGA
ATGGTAGGGAAGTTAAATATACAGTTCATGAATTCTGGAGGCATTACCTTAAACATGAAACAGAGCCCGAAT
AGTGTTTCCTCAGCAGAACGGCGTACCAATTTTATGGGCTGTCTGGGGTTTCCAGACATGGAAGATAGCTTA
TTCTGAACAAACAATTTTGAATTGGTATTTGATGCATCTTCCTTAAAGCTAACGTAAAATAGGAACTCTACGA
ATGCCCTCTCCCTAGTATCGGACATGAGTGCCTCAAAACATACAACTGCAGCCTTTCGCAGTTTTTCAATCTC

GCTCATAAAGGTTAGCTGTGTTGCAGGACGACAATAAGATTTTCCAACAGAAGCGTATATATCATTAACTGCT
GTTTTAACTACACTTGCGTCTCTGGACACAATAGTGTCAACTGAACAAGGGTTAGGTGTAGAATCTGGTAATT
TGATCACATTTTCTACCATATTTGCCCAATCTATTGATGACTTTATATCCATATTATTCGCAACACCATTAACAAA
AGCGTCGAATCTTGTAGACCTAGCAGAGTCGACCTTTGAAAAAATGCTCTCCATCCGCTTAGTCACATTGGC
AATGACAGTGGGGTTTTGAAAATCTGTTAGAGAAAGAGGTTCGGAAGAAATAGATGTGTCCATTGTATAATA
TCCACAAACAATGCTAGGCGTTTTGTTATTCATGATGCCTATATGCACCTTATTTTCTTCCTCGAGCGGGCTATA
TTGCCTGTACGCACCGGACAAACTCCCTGTAATATCTTCCATGATGGTTCTAATTTCATCAGCTGCCTGGTCGG
CTCTCTTGCGATTATTTTCTTGTCCAACGCGAGCAACAGACATTTTCAATAGGACATTATCCGATAGATATTTG
TCGTTTAAAGATGTTCTTTCTCCAGTGATGATAGAGGCCACAGCCGAAGCTGAAGGTTTTGGAGGTGCTCCA
TAGAGAGCAACAGAAAGAGCCGCAGCCTCCTCAGGTGATTGGATTTCTCCCTTGATTTTCTCAAATGAGTCA
GCTACATCCAGAACGGCTGAAGCAGTCTTCTTGAGGTATTCGTCCAATATTAAAGATGCAGACATTTTCCCCG
ATCTTTTTTTATCAAATTACGTGTACTGGTCTTGAATGCGATTTAGGAGATTCACATAGGGCCAGTTTACACCA
AATGAATTGGATGGTAGATTTTCAGCTTGATTTATATCTCTCTCGACAATGAGAGACGGTTCGTGTGAACCCC
TAAAAGTTAATGCCGTCTTTACAAGTTCAGCTACAGGCGCACACAAGAAACCTTTATTATCTGGACTATAAAT
TGCCCTCAAGTTTGGATTTGTCGATTTACACACAGCTTCTGCCTTTTTAAAATGAAGTACAGGATCAGTTAGA
AATTCAGGTCTTCCAGTCCTTTTCCATTTGTCGTAGTCTATAACATACCTGTATGTTTTGTGCTTACACGCAGGC
CCTGTTAAATATTCCGAGTAAAGACATGCACAAGTAGCCACGTACTCTCCAGAATCAGGGTCGCTAAAAATG
GATTCTGCCCTCCCTGCATTCATACTTTCATTGCATAAATCTTCACGCGTAGATACATCATGTTGCCTACTCAAA
ACCTGAGGAGATGTAGTTTCTTCAGGGAAAAGTTCCTTAAGGGCTAATTCTCTCAATTCGTTTCTTGACAAA
GGATATATTTTTTTCTGGATAAACCCTCTAGCCCTCAGGGGCATAGGGTCATGTGTGCCCGTTAAAACATCCA
CAATTTCACTAACGCTAAGAGATTGATCCTCTTTTTTCGCATCTTCTTCTTCTTTATTGTCCTCTTCGGTGCCAG
CCTCCTGTGTTATATCCAATATCCATTTCTTTGCCTTTTCATATTTAACTTTATCAATTTGTAAAAGTATAGAGTCC
AACTTCCTCAGTAGGTGCTCTCTCAATGATGAGGCCATCCGTTTATCTAAACCCTGGGCAAGAGTGTCCACAT
TATCCAGTTTCCCTATGCTTAGATGCATCGTGTCGTAGTACGCGGCTCGCTTATTCACAAACAAATCTCCTCCAT
TGTGATTGATAAACCACAAAAATGATTCAGGGAGTTCTTCCTCCAATTTTGGATTTTTCTTGATGATACCTATA
ATGTGGCCAAGATCATTGGCTGCTCTTTCTAGAGAAGACGCACTATAAACACTCCTGGCACATCTTCCCGACT
GTTGGTTACAAGAGAATTTTGTAACCTCCTTTCCGTCAACAGAGTACGTGTTCTCGCAAGCAGCGCCACAAT
CTCTGTCCGTCTCACAGTGTACATCAAACACCTCACAATCTGTCTTTGATACCACCCAGTCATGGAAGGCATTC
TTAGACAAATCAGTCGAAGACATTATTGTCTTTGTTCCTCCTTTCCACAAGTTGACAGAGAATTGATAGTCCC
TTTTCACTACCAATAATCTTGTTGAATATCTGTGCTTCAGTTTCAGTGGCTCCTGTTTCCTTGATAGCAGAAAC
TAATAGTTTATTCAAATTGGTTTTATTTTGAGTAGCTGAATGTAAGGAGGTTCTATAGTTGGCTGTGGGACGA
TCTTCCATCCCTCTCCTGCTTGCTAAGGATGCAAGAATATCACACATTTTAAACACTAAGCCCACCTTTGGAGC
ACCTGTTGTTTCGTCAGTGAATTTGTACACATAATCTATAAAATCACTAGGGGTGCATCTATTGTTCTTGTTAG
ACGTCTGAGAAGAAGAAGAAGAAGGAGATGAAGAGGAGGATAAAGAATCTACCAATTCTTGCTTCACTGG
CGGATGGGTTGCAGCTGCTTTAAAACGTTTAATCTTCTTGTCTCTGTTAATGTCAGATAATGCATTCTCTATGT
AACTGGTACTATTTTGATCGCAACTTGTACAAATACCCTTTATGATTTCATCACTACCCTTGTTCAAATTGACAC
CACACGCGTTGCACAGAAAAGAGCTCTTTTCTTGTCCGATTCGAGAAAATTTACGTTTTCTATTGTTACCTTT
ATTAGCAGTTTCTTGGTTATTTATAATGGATTTTTCAAATAGAGTACTAAAGTATGCATTTCCAGGTGTTTTATA
TGTACTTTCATTACTTGAAAGGGCCCTTTCGTTGTATGTTAGAGCATGTCCACGGGATTTAGGCAAGTTTGTC
ATTAAATGTAATAAACTATATACATCATTACCAAATAACTTGGCAAAATCAGGTGCAATGTCTGTTGAGATGTA
ATTATTCAAAAATCGGTACGACGCCATCATCGTGTTCAACCTGGCTAAAGTTACACGGTGTTTTTCCTGTAGTT
TTGCATTCCTCTTCGCCCTACTTGAACCCGACAAATAGTTACTATTATTGTTAATACTCATTTTGTTACCATTTTT
TTGATTACAGGCTCTCGCTAATAAAACATAAGGCGTTGGTATTTTTTCCTTCTGGTGCTTCCTCAAAAATGGG
GAGGCAATAAAACCCTTTGATTCACATTCCTCCACTATTTCTTCTGTACACAAATCCACAATATGATCAAGTTTA

TCCTTGTATTCGTTTTCATCTAGCCACCATTTCCCTTGACAAGAAATGCGACTATTCATCATACTGTTTGTATTTT
CAGAAACAGACACTAAACGTGCAACAGAAAACGGGTCACATCTCTCTTTACCGTAATATTGGTCATTTTTATT
CCACCACCTGTCTTGGTTTATAGGATCGAAAGAAAGTGTGTCTATAGCACGTCGAGTAGTTTCTTTTGAAGGT
TTACACAAATGTCCAAAAAGTTGTTGTGATCTTATTTTTTTAATGAAGGAGATACTCTCTTCTTTTGAAGGCAC
TAAATTTGCAGACATGGCAGCGTCCATACTGTTCTTTGTTCCATAGTATGTCCTATCCACTTCAGAAATATGTTG
TTGGTCCACCATCGCCCTTTTCACTTGCTCGGCCGATGGTTTGACGTACGGGTAGGAGTGGAGCAACATGTC
AATGTACAAGTACCATTCTAGATGCGGGATGCCGTATTTTTCTCTGACTCTTTGTGCTTCTTTGGGGGACATTT
TAATGTTGTTGTCCATAAAGTAATGGAATAAACCAAATAGTAATTTGGATACGACAGAAACATCACCGCACCC
ACTCTCTCCTTCTCCGAGCACAGTAATTTTCCCAACTTTAAATGTTAATGCAGTTTTTATCCTCAACCAGTCAG
ACATTTTAAAATTGGGATCCTTACTATCGCGCATCATTGCAACCTTTGTGTAGTATTCCCCTATAGTCTCTCCAG
GCAAGAGAGGCTTTGCGGGCACTCGCAGCTTGAAAAACATACCGGGGAACGTTTCTGGGTTATAACTGTAA
GAGATGCAATTTCTTTCGAAGAATTTCCCGAGTCGAATAAAGTTCAAGTGGAATCTATTAAAAGTGGACACT
GTATTCACTATAGACACGTTAGCCACACTGATGTTTTTCTGTAGAATATCAGCAATATCAGTCGTGGCTTTAATC
ACACACAATTTTGTAAGCGAAGGATCTTGAAGCCCGACAACTATCACTGATCCAGTTTCAAATATCAAAACAC
TGAAGAAAGATTCCCTGTTCTTCAATGCAGTGAACTGTTTCTGGGACGTGTCTCGCTTAGAAAGCACCATTC
TAGCTCTGTTGATGACCATTGATTTTCCTTCACTTTCTGATAGCATAGCCTTTTCTACCGCAGCAGCAGTGGCA
GCGGCAGCTGCGGCCGCGATGGCGTTTTCCTCGGTGGCACCTTTTTTTGTCTGTAGACCTTTTATTGTCTGTT
CGAATTCCTTGGTGGCGTATATGTCTTCGTCACTATTTTCCTTTGTTTCAGGTGCATCTTCCTTCTTTATTTTCTT
TGCTGTTTTCTTACCCAATTTCTTGTTCAATAACTGAATGTTTTGGTCTGTGATATTGTCCTTGAAATCGATGTC
TAGACTGACACAATTATTGTCAGTAGTACAGAAAGTGTAGTCGACATTTCCTAGGATAACATGCCTAGAACGG
CCTTTTGGAACAAGCACACCATGTCTCGTCATCTCGACGTCTGGTCCACTACTGAATGAACAGATGCGTAAG
AGTTTTGTTTTATAGCCTCCCTGAAACACAATTAATGAACTGAAAACCACTACAAAAGTAAGATGGAAAATCC
CGCTGAAGGAATGGGAGAAAGTATTTTCGATGCTGTAAGTCTGGCAACAAATAATCCTAAAAAGTCAAACTC
CAGAAACAAGAAGTTATTGCGGGAACTCAAGAATATGCGGAAAGATTTTCCTTCAACCTTTCTCCAGTGTCG
GATGATAGATTTTCACTTTTCTGGTGACATTATAGATAAACATTACTGTCATTCTGTAAACGTGCCAGATGTTG
TGCCTAATACAATATTTGCAGTTTTCTTACCTGAAGAGGACCGTGCCAATAACCCCGGGCTATACGATTCTATT
GAAGGAGTATGTATAACAGTCGAACAAGGTGAATTATGCATCATCAACAAGTCAAGCGTTCACGAGTTCAAT
ATTCTGGTGTCCTTGCATAAGGACTTATTTGGTGAAGATATCTTGGATGGAATAGAAACTGCATCAAGGGAA
GAATCTCGGTCTATCCACCTATATCTGGAGGCTGGGCAGAGTATCAGAACCCCAATCCCAAGACCAGAAGGG
ACAAATACTGTGAACTACACTATCGTTTTTTCTAACCAAGTGACGGTATAAAAGGACGAAGCAGGGCCCCAG
AGCATCATATCTCGTCTGGTAGTCGAGGAAGAAACATCTCTCGAGATGGACAATCTTATCACCAACGACAAC
ATTATCCTCGTCACTTTCCTGAGTGGATTGGCAGTCGGCTGCTCCATGACTATTGGGCTCGCACTGGCCATGA
ACATGCTCGTGAAGTGCATCGACAGAACTACTACTTGCATTTCTTGCTCACCGTGGGAGAAGAATAAGAATA
AGAAGAACAGGAACGGGAGCAACACCGAATCCAGTTTCATCAGCCACGTCCGGTTCAACACTCCAGATAAG
GACCTGGACATCTCTGAACCCATGCTCAAATCTACCACTTACGATCTGGCCAATGTTACCCCTCAAGTCACAA
AACTGGTAACATTTTCTGGTCCAACCTATGCTAGTCCGCCTACACCCAGGCCAGTTGCCAATACACCTCAACA
ACAACCAACAAGTACAAATAAAGAGGAAGAAAGTGTCTATATGCCAATGTCGAGCTGCTCGTCGTCATTTTC
TTCTGACAATAGTCTTCCTCTGCCAACACCGCCGCCATCTCCACCTAGAAGCAATGGCGGTGATTACGTGTCA
TATGTAAACGGACGACATCTGAAGCTTCCTTCAAACCCACCTTCTCCCATCTTCAATATCAAGAATGAGGAGG
GAGAGGATGATAATGTGGAAGAACATGTCTACGAATACGTGCCAGAAGTACCTCAACAATCTCCATCTATCCA
GAAGTGTATCCAGGAATTGAAGGAGATGAAACACAAGAAAAACACCCTAACCAGGAGCAGTAGTAACAAC
AACAACAATGCTCCACGTATAACCCAAGTTACGTTTAAGAAATTCCCACCTAACAATAATAACATGTGGGAGA
ATCATGTGTATGGAAACACTACAATTGTGTCTTCCACACCTTCTCCTACCTTTATTCCTTCACCTAAAAGTATCA
TAAGGAAATTGTCATTTAAGAGGAAACAATAAAATATTATACTTTTAGGTGAAGGAATAAAG

>Unigene33511_All pri-miR(wssv-m7) size 1896
AGGCATGCAAAGAAATTAAAGTTTATTGTCCATTATATTCAACAAACTTTAATTTCTTTGCATGCCTTGTTCAA
AAGGGCAACATAATCGCCTTCTTTCTCCTCCCTCTTTCTCTTTGTTCCTCCACTGCTTGTGGCTACTTCTTCTTC
TTCCCCACGTTCCTCGTTGTACGTCTCAATTGCCTTAACTGTGTTGAAAATCATGCGACGCTCTCGGCGCAGA
CGCTTCCTTTCCTCCTCTGGTAATGTTTCTTCCTTTTTTATTATGTCCTTGCCTTCTTTAGAAGAGGGGTCAATC
ACTTTTATTTCTACACCATCTTGATATACTTTTCCATTATCCATGAAGATTTTGATGTCTGATCCAGTAACCTCTC
TGACCATCTCCATCAACAACCTTTCATTCGGAGGATCGATGAATGATATTTCATTACCCTTTCCGGATGCAATG
AAATGGTTATTAAAATGGTTTCGTATACTATCTCTGATGCATATAGGTGCATCTGAGCCAGTTATGGACATGAC
CAATCTGGGCCTACGTTCATCCACGTCCCTCTTTCTGTAGTCGTCCGAAGAGACGTATCTGATTCCTTCTTCTG
TGGCAGAGTAAAAGGAACCATTTCCATTGGTTCTGCACAACAAGGAGAAATCTCGGTCAGAAAAGGCAAA
GACCAGACAGGCACCGCCGTCATATCTGGATGATGCAATTGAGGACTTTTTATGCACATCTCGAAGAGAAAT
CCTTTGTTCTTCTGAGGCAGCATATAGCTTTTTGGATTCTTTAAGTTTTTCTTCCAGAACACTTCTTTCGCTAA
CTAGAAGGTTGACTTCATTTTTTGCAAATCTGATTTCCTTTTCGAAATTATCTAATTTTTGATTACTCTTTTCTA
GCGTGTATACAACTGCAGCATTGAAAAGGCATGACATGTAATTTCCAATGCTTTTAGCTGACGGGTGTGTGCT
GTTGTAGAAAAGTAATCCAAGTTTATCCATTGGTACAATTAGGCACATATTTGAAAAACTGCCAACTATGGGG
TTTGCGATACTTAAGCCATTGTACGTTAACCTATCACAGATATCATACACTGATACACGAGAAGAGGTACCACC
GTGTTGCACTAACACTTGTTCCATATTCTTAAGAGTACGGGTACAATTTCTAGGAGAAAACCCACACCCTACT
AAGAAGTCTGTTGTGATTATCATATAATCACATTTCTTAACGGCCGCATTTGAAAGACGTTCATGTATGATTCT
GTATAGGGGTGAAAATTTAAAGTTCTTGTCATTAACTAGAGCCTTTTCTTCCACAATTTTGTTCATTTCCTGGA
ATGGGCTTGGTAGTGATTCGTTCCATTTTTTTCCATAATTAGGGTCTTCATCTTCTAAGAAACTACCTTGTTTT
GGGAGACTGGATGTGTATTTTAGTCCAATCTGAAGCGTAGGATCTCCTGTTAATTGTCTCAATCTTTCGTTACC
GTTCTTGATGTGGAGTACTTCCTTGTCTCTAAGACGAACTATAAAATGTCCTTCCTTTAGCTCACTTCTCCTGA
CACTCTTGACTGATTTGAATGTTAATAGTACCCGTTCATAATTTTCGGTATTTTCCCCAGACAAACAAATACCAT
TTTTTACTTTTAATTGTTTAAAGTCTTCCATTTTTATACCGTGTAACCCTAACAATGGTGTGCCAGTATAATAAAT
CTACGGACCATGCTCTTGATCTGGGAAGTGTACTCGTGATTTCTGTAGAAATTGCTATTTCCTATTCATTATTTC
CCATTACAGGAAAAGATGATAAAATGAAGACGTGCGATGAGATATGTGTTTTTTCAATATCGTATATCAAATTT
GGGCAAATTACATATTTTTTAGCGTCAACACTCATCTTCATGCAAGA
>Unigene33576_All pri-miR(wssv-m1) size 2319
CACTCTTTTTAAACTGAACGCCAAAACTATTATCGATTAGAGGAATAGTTTTGGACACGTTATCAATATCATTT
ACAGCACTGAGCATTTCTGGAGATGCTGGTTCGATTGTTTCGATTTTCCAGTACTTTCCGCTATTGATATTACG
TTTCTTCTTTGGGGGTTTCGTTTGTGGTTGCTCTCCTCCTTCATCATCTTCACTATCCGAAGCTGAAGAAGAA
GATGAGGAAGAAGGAAGGGGTCTCTTGACACCTCTAGGGGCAACTTCTGCTCCCTCCACAACACCTTCAAC
AGTAGTTTCCAGAATCATTTCTTCTTGGGTATTATTCTCAACAAGAGACATGTTGTTGGTTCTGGGGTTGGTT
GGTTCAGTTGGGATATTGAATGATAGACGGGTGGATTTTTAGATACTTTCCATTGACCATACTGACACTTTGT
TATAATTTGGGTACAAGGGGACTCTTACATCTTGGACCGTGAACGACCCAAAACCACAAACGCTTAAAGTTT
AAAAACGACCACATAATACTACATTTTTATATGAAAATTGGCACTTTAAGTTCTGAAGCTAGACATCTCAGAA
AGTTTATCCATCATACTCTTGTCGTCTTGTTTTATCGTGTTTATGCCAGTTATATTAGATCCATATCCTAATGTCCG
GCCGGCATTACTGGGTGTAGTAGTAGTAGTATTTTTGTCTTCTGGAGATATCATGTTTAATATCCAATCTCTCTT
CATACGTTCCACATGATCCTTTGTCTGTTCAGTTCCTGATCCTAATACTCCTCCTCCTCTTCCTCTTGTCTGTTCC
AGAAATGCTGAATGTGTTGGTTGTTGTTGCTGCAACATCAGGGACGATCTGTCTCCTCGTCTGCTGCTATGAC
TGTTATTTGCAGTGTTTAATACACTCATAAAATGGTTGTTGGCGAGCTTTGACATGTCGGCGGCTGCACTCAC
GGCGATGGTCGTACACAGGTGAGTAGTATTACGTATCATTCCAATTGTATTTGTTATATTAATAGCATTTGTAGA
TTTTGATGTTGCTTGATAATTTGCTGCTCTCATTTCAGATTCCGCTGCTTCTGCTTCCATGAGCTTGATTGATGC
TGCGCCTATAACAGCTACCCCCATGGCGTTTGCTAGCTGGGAATGCACAACACTTGTAGGCTTATTTGTAACC

TGTTCGACGAGTGCTCTTTCTGCTCTATTTTTATATAAATCTTGTGTATTTTTATCATTGTTGAATTTTTTCAAGT
ATCGATTTGATACATCTTTCATTGTATATTTTGCTAGAGGGGTGGGTTCACAGTCACTGCCATTTTTTTCAAAC
ATGTAAGAAAGAGCACCGTCTACAAAAGATCCTCCTTCTAATACATTAGAAGGGTTCATATTCGCATTATTTGT
TAAGGCTTCAGACATTGATTTTTCCAGAGACTGAGCGGCAAAATTAACTGCCATGTCCATAGTATTTGAGTCC
ACTAGTAAAGTGTACGCTTTAACTCCTCCTTCATCAAATTCCCTCGAGCGTACGACCCCGATACATCCTTCTGC
GATGGAATAAAATCCTCGTGCCAGATTTGACATTGTAGTCATATCCTGTAATTTTATAGATGAAGGATCGTTTC
CGGGAAAGATAGAAGTTTCTTTTGATGTTGGAGGAAAGAGTAGTATGGACATGGCTGCTGAGTTTTGTTGG
TCCATCGTATTGTTTACGAGCGTGTTGGCATTTTTGATTATATTATTAGCTTGGGCACTTTGGGATATGTTTTTC
AATAGGTTGGTTAGCCTTAGACCAGTTACAGTATTATTCCCTACAGATTCTGGGAGGTTAGAAAATAGTATATT
GGGCGAGATATTAGATCCGATAGATATAGCCGTTTTTATCTTGGCCGCCAAATTTATGCGTCTTCCCATAGGCG
AGTAGTTTTGCTTGAACGCGTCAGCCGTTAATTGTACACTCTTATTGGAAGGGAATGAGTTCACTATTGATTT
TCCCATTTCTAGAAATACTTCTCCCACCACTCTTTTCAAATCCGGTTGTTGTTCATGTTGAGGGCATTGTTGTT
GTTGTTGTTCGTCTTCGAACGGCATCACCGACATCATTTCTTCATTCGAGGTAGAGACTTTGTTACTAATTGTT
AAATTTTTAAAGAATGACTCTACAACAGTTTTAAGTCCTCTATTTTCATTCGAGGGTAAAAGTTTTGATGTTAG
AATGAATGCTTCATGGCGATTAACTACAGTATTATCCACCACCACGAAAAAGAAGGCAGCTAGCCTTGCCAAT
TCAGCTCCAGAAT
>Unigene39471_All pri-miR(wssv-m11) size 2795
TACCGCCTCCCCATTCCCTCTTTCCTTTCCCCTTCTCTCCCTCCCTCCCTCTCCTCCCCTTTCTCCCCTCTCCTTG
CTTTTCTCTCCCCTTTCATTGCCTCCCCACCAATCTTGTCTTTTTCGCTACCTCTTCCTTCCCCTCGCCTGCTTCC
TCTCAATCTTATTTCTTCTCTCCCTCTCTCCCTCCCTCTTCCCTTCCTTTTCCCTATTTTAACCCTCCTCTTCCTCT
CTTCTCGCGTCCCTTCCCTTTCCCCCTCTCCCTCCTTTCCTATTCCATACTTCGTCCTTTATCCCTATCCTCCTTCT
TTCATTATCCTCCTTCCCCTTTCCTTTCCCCTTCCATCCTCTCCTCATCTCCCCTCCCTATCCCTCTCTTCCACCTC
TCTTCCTTCCCCTCGACCTGCTTCCTCCTTCCTTCCTCGCATCCTTTTCCTCCCCCCTCCCCCCCTTCCTTATCTC
CCCTCACCATTTTTACCCTTTCCTTTCCCCTCGTCCTCCCCTCTTCCCCTTCACATACCGCATCCTTCTTCCTCTT
CCTCTTCCTCCACATTAGAAGGTACGAGGGGTGTTGTCGGGGCAGCAACAACAGATGTAGGCCTGAAGAAT
TCTTCCTCTGTTCTCTCACACACCTCATCATCGGAATCAATCTCAAAATACAGGGTCTTCTCGGCTTGTACTCTA
TTATTCTTCTTTTCTTCTGCTGGATTCACCTTGATGATGTTTTCGTCCTCAATTTTTGAGACTCCCTTTACGCCA
TCCTTTGTCTTGTACATCAATTCAATGATTGTGTCACCGTGAATATCATCACTAGAGGCGGATGTTACCGTCAC
AATATTTGCAAATGGGAATGCACTCTGTCCCTCTTCCAGTTTATTGTATTGATTAAGTCCTGTGCGGAGAAGTT
GTTTGGCGTATAACTCACTGTGCTTTTTGCACCCTTCTGTGTCATACTTGATAAACTGACAATTTTCGTATTCA
GGCAGGTCCCAGTCATCAAACAAAGTCCCCGACTTCAATCCTGCGGCAACCTTTGCGTATACAGCCATAGGA
GGCACAAGTTCTTCCACTTCCGGATGTTGGAATTGCTTTGCAAACATATACTTTTGAATCCCCTTAGCGCACA
CCTTTACAATGTCAATACCCATGAATGCCATCAAGTTAGCTGGGAGTCTAGGTTTCTTCCCATTCTTGTTACCC
CAGCTCTTGTCCAGATCAGGGTTAACATTATCGTGGAACAGAAACTTTGACTTGTCATTATAATAAACAGTCC
CCTTAATTGACGCTTCATACACGGCCCTGGCGGCTGAGGCATAGTTTGAGCGAGTGAGGATATAGTTACGGA
AGCGTTTAACGCCAGCAGTATCCCCCTCTACAAACTTATCCCATGCAGTCAAAAATCCCTTGCAAAACATTGA
ACGGTCATATTTGGAGGGGTTAATCTCAGAGATGAACACATTAAAGGTATGAATTTCTAGGGGGGTAAGGG
CAGTTTTACGGCACATTTTACTCAAATCTTCATCTACATTTACCTCTTCTTTAGGGATGGTGCTCTTACTTGTAA
GAGTTTCAACTTGGGGAAAAACAAAGTCATCCAAATTAACATCAACAGGGGCACCGGCAAACATGCCATGA
TTGTCCCCATCTACATTATTAAAGGGATTTTTGGCTGTGGCTGTAACTTTCTTTGATCTTCCGCCGCCACTGTT
ATTACTACCACCAACACGCTTGTTACTGATGTTGTTACTATTATTAGAGGTCGAGGGTTTGTTGTTGTTGTTAA
CAACAGTTGATGGGACACTATTATTATTACTTGGAGCCACATCAAGTGATCCAAAAACAACATGTCCAAACAA
CTGGTTAAGTACGTTCACATTAGACACTTTAGTCACCAAAAACTTGAACACTTGGATTATAAACAGGGTGCA
AACTAAGACTGTCCTAGAGACAGGGGAACTATTACTATTAGAGCCACTACCACACTCTTCAAGCAAACTCCA

CACGTTTTGAAACATACCAGAAATTTGAGCGGAAGTTGATGAGTCGACTTCTTGTCCAGAGGGGAGGCTTG
AAGGGTTGATGACGTCATCAGAGACTGGAGAGAAGTTTGCAGACACTCCCATTTCTTCGTCCTCACTCTTCA
TAAGGGCGAACATCTGCCTCTCAGTCACGGGGCGTTGGGGCATACTTGCACACAAGGCAGAGTAGTTAAAA
TCGTCTCCTTGGAACTCCATGGCTTTAACTTCTCTCATCACAATGTTGGTGGTGAAGCGGGTCTTCTGGCAAA
AGCTCACCAGTGCCTTCACAACCTTCGCACCAACACAAGCAGCCGGGGACGAAGTTTGGGCGCCTTCGGG
AGATAGAGGTCCGGACATCACCTTATCCAAGAGATCGGCTGCAGCGTTCAGGTGAGGCGGGGCCATTGTGA
AACATCCATAGTCTCCGTACTGGTTGGAGATTTCTGTCAAAGAGTAAAGGAATTGGACTTAGAATATTTTTCA
CTTCAACCAACAAAATATTCAGCATAACATAGAAAATTTCAAAGTCCAAAGTGTATAGTGAGAACTTGTGCTT
ACCTTGTATGAATTGGAAGATCTTCTCGTGCTCCTCGAATAGATGGAGCAAGTGTTTCTTCATATCAGACCGG
CGAGAGCTAGTCGAGACGTATGAAGTTAGTTCCTTTAGTTCCTCAGAACTAAGTAACTTAGAGTCCAACTTG
GTGTAAGGACTTGGTGCGGCGGGGGCTGGAGAGGCCATTCTTGTAGCTTGAGTATTGGCAGGTCTCTTCTA
GGTAGAAG
>Unigene33576_All size pri-miR(wssv-m12) 2319
CACTCTTTTTAAACTGAACGCCAAAACTATTATCGATTAGAGGAATAGTTTTGGACACGTTATCAATATCATTT
ACAGCACTGAGCATTTCTGGAGATGCTGGTTCGATTGTTTCGATTTTCCAGTACTTTCCGCTATTGATATTACG
TTTCTTCTTTGGGGGTTTCGTTTGTGGTTGCTCTCCTCCTTCATCATCTTCACTATCCGAAGCTGAAGAAGAA
GATGAGGAAGAAGGAAGGGGTCTCTTGACACCTCTAGGGGCAACTTCTGCTCCCTCCACAACACCTTCAAC
AGTAGTTTCCAGAATCATTTCTTCTTGGGTATTATTCTCAACAAGAGACATGTTGTTGGTTCTGGGGTTGGTT
GGTTCAGTTGGGATATTGAATGATAGACGGGTGGATTTTTAGATACTTTCCATTGACCATACTGACACTTTGT
TATAATTTGGGTACAAGGGGACTCTTACATCTTGGACCGTGAACGACCCAAAACCACAAACGCTTAAAGTTT
AAAAACGACCACATAATACTACATTTTTATATGAAAATTGGCACTTTAAGTTCTGAAGCTAGACATCTCAGAA
AGTTTATCCATCATACTCTTGTCGTCTTGTTTTATCGTGTTTATGCCAGTTATATTAGATCCATATCCTAATGTCCG
GCCGGCATTACTGGGTGTAGTAGTAGTAGTATTTTTGTCTTCTGGAGATATCATGTTTAATATCCAATCTCTCTT
CATACGTTCCACATGATCCTTTGTCTGTTCAGTTCCTGATCCTAATACTCCTCCTCCTCTTCCTCTTGTCTGTTCC
AGAAATGCTGAATGTGTTGGTTGTTGTTGCTGCAACATCAGGGACGATCTGTCTCCTCGTCTGCTGCTATGAC
TGTTATTTGCAGTGTTTAATACACTCATAAAATGGTTGTTGGCGAGCTTTGACATGTCGGCGGCTGCACTCAC
GGCGATGGTCGTACACAGGTGAGTAGTATTACGTATCATTCCAATTGTATTTGTTATATTAATAGCATTTGTAGA
TTTTGATGTTGCTTGATAATTTGCTGCTCTCATTTCAGATTCCGCTGCTTCTGCTTCCATGAGCTTGATTGATGC
TGCGCCTATAACAGCTACCCCCATGGCGTTTGCTAGCTGGGAATGCACAACACTTGTAGGCTTATTTGTAACC
TGTTCGACGAGTGCTCTTTCTGCTCTATTTTTATATAAATCTTGTGTATTTTTATCATTGTTGAATTTTTTCAAGT
ATCGATTTGATACATCTTTCATTGTATATTTTGCTAGAGGGGTGGGTTCACAGTCACTGCCATTTTTTTCAAAC
ATGTAAGAAAGAGCACCGTCTACAAAAGATCCTCCTTCTAATACATTAGAAGGGTTCATATTCGCATTATTTGT
TAAGGCTTCAGACATTGATTTTTCCAGAGACTGAGCGGCAAAATTAACTGCCATGTCCATAGTATTTGAGTCC
ACTAGTAAAGTGTACGCTTTAACTCCTCCTTCATCAAATTCCCTCGAGCGTACGACCCCGATACATCCTTCTGC
GATGGAATAAAATCCTCGTGCCAGATTTGACATTGTAGTCATATCCTGTAATTTTATAGATGAAGGATCGTTTC
CGGGAAAGATAGAAGTTTCTTTTGATGTTGGAGGAAAGAGTAGTATGGACATGGCTGCTGAGTTTTGTTGG
TCCATCGTATTGTTTACGAGCGTGTTGGCATTTTTGATTATATTATTAGCTTGGGCACTTTGGGATATGTTTTTC
AATAGGTTGGTTAGCCTTAGACCAGTTACAGTATTATTCCCTACAGATTCTGGGAGGTTAGAAAATAGTATATT
GGGCGAGATATTAGATCCGATAGATATAGCCGTTTTTATCTTGGCCGCCAAATTTATGCGTCTTCCCATAGGCG
AGTAGTTTTGCTTGAACGCGTCAGCCGTTAATTGTACACTCTTATTGGAAGGGAATGAGTTCACTATTGATTT
TCCCATTTCTAGAAATACTTCTCCCACCACTCTTTTCAAATCCGGTTGTTGTTCATGTTGAGGGCATTGTTGTT
GTTGTTGTTCGTCTTCGAACGGCATCACCGACATCATTTCTTCATTCGAGGTAGAGACTTTGTTACTAATTGTT
AAATTTTTAAAGAATGACTCTACAACAGTTTTAAGTCCTCTATTTTCATTCGAGGGTAAAAGTTTTGATGTTAG
AATGAATGCTTCATGGCGATTAACTACAGTATTATCCACCACCACGAAAAAGAAGGCAGCTAGCCTTGCCAAT

TCAGCTCCAGAATCCACCATCATTGTAACCAC
>Unigene39453_All pri-miR(wssv-m9) size 1045
GCGTGTGCTGGTACAGGAATATTTCTGCGGTAACTTTCCCATTTCTTACATTGGGCAGTGACCTCCCCCAAGA
GTGTAGTTTTCTTCTCCAATATAGATTCACATGCTCCAGGGGAAAGCTTAAAAACATTCTTTGCCATGTGATCG
ATGCTCATGGTTTTTATCCACCCTTTACCATTATTCTTGCATAACCTGTTCCAGTTCAAGAGAGGGCTAGTGTC
TAAAACGGATAGGTTTTTACTACCAAACCCAACAGGCCACCAAGATGAAGGGGGTTGTTTAGTACGGTTGT
TAGACCACAAAAACTTGGACGCTTTTTCCTTTGCGTCAATACAGCTCTGTTTTATGGTCTCGTAGTTGGACGA
CACATCTTGCACGTCAGAAATGAGGCCTAATTTTGCGGCCTGTCTAATCAAATGGAACGGAGATTCTAGACC
GGCTGCAATATTGGTATCAGCCTCTGCCACTGCAAAGTAACACGGGGGTTTTTCTAGCTTGACCCACTTGTC
AATGGAAGAAAAGTTCCTCTTTACTACATTTGTTTCAGTCACGGTATCACAAGAAGATTTCAAGTGTTTTGGC
CCCTTGACATGGGAACGGAGGACAGACAGTGGAGTATAGTCTCTAGCCTTCAAATTGACAGTAGAGAATTT
TGCACAATTTGGATGAGAGAATCTTTTTCTGTACTGGGTGTCGGCATTGGCGCGGTGCTTCCTCAACACAGA
CGCGGCACTAACAGAAGGCAATTGCTGCTTCTTGATAATGTCTGTCGCATACGTTGGATTTAATGTTGCAAAT
TGATTTTCATCAATAGTAATAGGCCTCTTCTTCTTATTTCTATTAAAGTTGTAAGGTGTGCTCTCGTAGTCCTCG
CGGAGCCTCAAAGTCACAGGAGGCGGTGCTTTTCCTCCCACCTCTCCATTATTCTTCTTTAGTCTCTTCATGG
GCCTAAGAGAATAAGGAGAATCTGAGGGAGGGGATCGGCGAGTAGCGTTTTTAGAGCAACAAGATTTCAC
AGCTATGGCTGAAGGTATCTTGAATTGCC
>Unigene37970_All size pri-miR(wssv-m8) 1855
CAAGAGAAGAACGGCTAAAGACGTCAAAAATACGTTTAAACAAAGCTCTTAGTGATATTGTTGAAGCCACA
AACGAGCGTGTTGATGCGTTGAAAGAGAACCAAGCATTAAATACAGAATATGACAAGAAGGATAATTACTTC
CAGGTTTTAAAGTGCTCGATAACACCTTCTGTACCAACAGCTATTATAGGCGCACACGTGAAACAGGTGGCC
AAAAGTAGCGAAATCGAACTGGCCGTGAACGAACTCGATATAAAAAATAAGTGCTCTTTAGTGTACAACGAA
AATGAGTCGTTAAAATTTTTCAGGGACCATGAGAACCTTATACTACAAATTGCCGTCCAGTTATTCTCTAGGC
ACGATAACACCAAATGCGTGGGGGCAGAAATATGTGTTAAAGGCAACGAAAAAAACAAGTTTGTTAACAAA
CTGGTGGTAAAAAAACTCCCCAATGCACCATCCTCATCTTCAACTGTGCTAGAAATTAGAGGCGCTACCAGA
AATTTACTGGAGAATAATTTCAACAAGGGAGAAAATAACACTGTCAATGAAAACAAGGACATTCCTCCTTCA
GAACGAGCCAACCTGGACACGACCAAGGCAGAAATATCGCACGTCTTTTCCACTCTACACAGACTGGACAC
TAAAAGGAAGCTTTTCTTTAAAGGCAACACTTTTTATCAACGAAAACCAACATTCGATAATAAATTCAGGTGG
ACAGAAGTTATAGGGTGGACAGAAAGTGAAGCATCAAAACAAACCACTAAATCGCTAGACAAGCCAACGG
ACGACAATTTATTCGTGCTACCCCATTCTTTCAATAATTTGGCAGACCACTTACGTTTGAAATTTAAAAACGTC
CTCTATAAAAATAGTACCGCACATCCCGGCAAACGAAATTACTACAAGACTCAAGAGACGCTAATAAATCCCC
AGATTGATTCGGCGAAAGAGTACAAGATGGTCTTTGCAGAAATCGACAAGTGTTTGGATGTTCTTTTGGCCA
TAGGGAAGAATGACAAATACACAAAAAGCACTGTCATACAATATAGAGGAAAGTTTAGAAGGTATTTAATAT
TCTGCTACGCCTTTTATGCTCTAAATAAGGCAAAACATTCTCGCGCAGTATCCCCTCTACCATTTAATTTCTTTA
ACCTTTTCTCCTTCATGTATTGTCATGGTCCGTTTCTCCATTCCGCCAGTTTTTTGTCCACATTGACGTTCGTCT
ATCAACACATGTTTTTCCCCATGGGCACAGCCGCCCCATCCGTCTCAGCCAAGCGGCTCATGGATATCGATTC
CGCCCTAATGAAAGGAGGAAAGGGGGTGGGTGTGAGGGATTTGGTTCACCTTCAAAAACAAGTCTCCAT
ACAAGAACATTGGTGTCTTTCTTAGGTTTTGCTGAAATGGCTATGGGAACAATGACGGCTCTCTTATCTGGTG
TAGAAGTGCGTGTATCTCCAGCTCTCCAACAAAGGATATCTAAATCCCTAGAAAGATGGTGTGATTCAGTCAT
CTTTATATATTTCACCTTTGTTTTATTCCACAGATTCAGTGGTGCGAAAAAAGTATCACTCGAATCGGCGCTTC
GCCTCATCATGGGGCAGACGCACGCCCACACAAATAAGGTGAGGGCCGCCAAGAGATGCCGAATAGAAGC
AGCGGAAATGGAAGGTGTGGAAGAAGAAGAGGCGGGCCTGACACTCTCTTATGCCCATCTATTGGGTCTTC
CTTACTCTATACAAAAAGCCCTCGGATTACCTGTCCCTAAGATAAACCCTCTCATGACAGCATCTTCTTCTCAAT
ACAATTTAGGGGATTTTGTAGGCGTGGAACAACTTCTAAAGGCTAA

References

Aliyari, R., Wu, Q., Li, H.-W., Wang, X.-H., Li, F., Green, L.D., Han, C.S., Li, W.-X., and Ding, S.-W. (2008). Mechanism of Induction and Suppression of Antiviral Immunity Directed by Virus-Derived Small RNAs in Drosophila. Cell Host & Microbe *4*, 387-397.

Brackney, D.E., Beane, J.E., and Ebel, G.D. (2009). RNAi Targeting of West Nile Virus in Mosquito Midguts Promotes Virus Diversification. Plos Pathogens *5*.

Bronkhorst, A.W., van Cleef, K.W.R., Vodovar, N., Ince, I.A., Blanc, H., Vlak, J.M., Saleh, M.C., and van Rij, R.P. (2012). The DNA virus Invertebrate iridescent virus 6 is a target of the Drosophila RNAi machinery. Proceedings of the National Academy of Sciences of the United States of America *109*, E3604-E3613.

Chotkowski, H.L., Ciota, A.T., Jia, Y., Puig-Basagoiti, F., Kramer, L.D., Shi, P.Y., and Glaser, R.L. (2008). West Nile virus infection of Drosophila melanogaster induces a protective RNAi response. Virology *377*, 197-206.

Flynt, A., Liu, N., Martin, R., and Lai, E.C. (2009). Dicing of viral replication intermediates during silencing of latent Drosophila viruses. Proceedings of the National Academy of Sciences of the United States of America *106*, 5270-5275.

Han, Y.H., Luo, Y.J., Wu, Q.F., Jovel, J., Wang, X.H., Aliyari, R., Han, C.G., Li, W.X., and Ding, S.W. (2011). RNA-Based Immunity Terminates Viral Infection in Adult Drosophila in the Absence of Viral Suppression of RNA Interference: Characterization of Viral Small Interfering RNA Populations in Wild-Type and Mutant Flies. Journal of Virology *85*, 13153-13163.

He, Y.D., and Zhang, X.B. (2012). Comprehensive characterization of viral miRNAs involved in white spot syndrome virus (WSSV) infection. Rna Biology *9*, 1019-1029.

Huang, T.Z., Cui, Y.L., and Zhang, X.B. (2014). Involvement of Viral MicroRNA in the Regulation of Antiviral Apoptosis in Shrimp. Journal of Virology *88*, 2544-2554.

Huang, T.Z., and Zhang, X.B. (2013). Host defense against DNA virus infection in shrimp is mediated by the siRNA pathway. Eur J Immunol *43*, 137-146.

Jayachandran, B., Hussain, M., and Asgari, S. (2012). RNA Interference as a Cellular Defense Mechanism against the DNA Virus Baculovirus. Journal of Virology *86*, 13729-13734.

Leger, P., Lara, E., Jagla, B., Sismeiro, O., Mansuroglu, Z., Coppee, J.Y., Bonnefoy, E., and Bouloy, M. (2013). Dicer-2-and Piwi-Mediated RNA Interference in Rift Valley Fever Virus-Infected Mosquito Cells. Journal of Virology *87*, 1631-1648.

Ma, M.J., Huang, Y., Gong, Z.D., Zhuang, L., Li, C., Yang, H., Tong, Y.G., Liu, W., and Cao, W.C. (2011). Discovery of DNA Viruses in Wild-Caught Mosquitoes Using Small RNA High throughput Sequencing. PLoS One *6*, 7.

Mueller, S., Gausson, V., Vodovar, N., Deddouche, S., Troxler, L., Perot, J., Pfeffer, S., Hoffmann, J.A., Saleh, M.C., and Imler, J.L. (2010). RNAi-mediated immunity provides strong protection against the negative-strand RNA vesicular stomatitis virus in Drosophila. Proceedings of the National Academy of Sciences of the United States of America

107, 19390-19395.
Myles, K.M., Wiley, M.R., Morazzani, E.M., and Adelman, Z.N. (2008). Alphavirus-derived small RNAs modulate pathogenesis in disease vector mosquitoes. Proceedings of the National Academy of Sciences of the United States of America *105*, 19938-19943.
Nandety, R.S., Fofanov, V.Y., Koshinsky, H., Stenge, D.C., and Falk, B.W. (2013). Small RNA populations for two unrelated viruses exhibit different biases in strand polarity and proximity to terminal sequences in the insect host Homalodisca vitripennis. Virology *442*, 12-19.
Sabin, L.R., Zheng, Q., Thekkat, P., Yang, J., Hannon, G.J., Gregory, B.D., Tudor, M., and Cherry, S. (2013). Dicer-2 Processes Diverse Viral RNA Species. PLoS One *8*, 16.
Schirtzinger, E.E., Andrade, C.C., Devitt, N., Ramaraj, T., Jacobi, J.L., Schilkey, F., and Hanley, K.A. (2015). Repertoire of virus-derived small RNAs produced by mosquito and mammalian cells in response to dengue virus infection. Virology *476*, 54-60.
Schnettler, E., Tykalova, H., Watson, M., Sharma, M., Sterken, M.G., Obbard, D.J., Lewis, S.H., McFarlane, M., Bell-Sakyi, L., Barry, G., *et al.* (2014). Induction and suppression of tick cell antiviral RNAi responses by tick-borne flaviviruses. Nucleic Acids Research *42*, 9436-9446.
Scott, J.C., Brackney, D.E., Campbell, C.L., Bondu-Hawkins, V., Hjelle, B., Ebel, G.D., Olson, K.E., and Blair, C.D. (2010). Comparison of Dengue Virus Type 2-Specific Small RNAs from RNA Interference-Competent and -Incompetent Mosquito Cells. Plos Neglected Tropical Diseases *4*.
Siu, R.W.C., Fragkoudis, R., Simmonds, P., Donald, C.L., Chase-Topping, M.E., Barry, G., Attarzadeh-Yazdi, G., Rodriguez-Andres, J., Nash, A.A., Merits, A., *et al.* (2011). Antiviral RNA Interference Responses Induced by Semliki Forest Virus Infection of Mosquito Cells: Characterization, Origin, and Frequency-Dependent Functions of Virus-Derived Small Interfering RNAs. Journal of Virology *85*, 2907-2917.
Wu, Q.F., Luo, Y.J., Lu, R., Lau, N., Lai, E.C., Li, W.X., and Ding, S.W. (2010). Virus discovery by deep sequencing and assembly of virus-derived small silencing RNAs. Proceedings of the National Academy of Sciences of the United States of America *107*, 1606-1611.
Xu, Y., Huang, L.Z., Fu, S., Wu, J.X., and Zhou, X.P. (2012). Population Diversity of Rice Stripe Virus-Derived siRNAs in Three Different Hosts and RNAi-Based Antiviral Immunity in Laodelphgax striatellus. PLoS One *7*, 11.
Xu, Y., Huang, L.Z., Wang, Z.C., Fu, S., Che, J., Qian, Y.J., and Zhou, X.P. (2014). Identification of Himetobi P virus in the small brown planthopper by deep sequencing and assembly of virus-derived small interfering RNAs. Virus Research *179*, 235-240.
